# Cell autonomous angiotensin II signaling controls the pleiotropic functions of oncogenic K-Ras

**DOI:** 10.1074/jbc.RA120.015188

**Published:** 2021-01-08

**Authors:** Daniela Volonte, Morgan Sedorovitz, Victoria E. Cespedes, Maria L. Beecher, Ferruccio Galbiati

**Affiliations:** Department of Pharmacology & Chemical Biology, University of Pittsburgh School of Medicine, Pittsburgh, Pennsylvania, USA

**Keywords:** caveolin, senescence, Ras, angiotensin, oncogene, ACE, angiotensin-converting enzyme, AGT, angiotensinogen, Ang I, angiotensin I, Ang II, angiotensin II, AT_1_-R, Ang II receptor type 1, ChIP, chromatin immunoprecipitation, DPI, diphenyleneiodonium chloride, HMGA1, high-mobility group AT-hook 1, JAK, Janus kinase, KLF6, Kruppel-like factor 6, MEFs, mouse embryonic fibroblasts, NHBE, normal human bronchial epithelial, NOX2, nicotinamide adenine dinucleotide phosphate oxidase 2, NSCLC, non–small-cell lung cancer, OIS, oncogene-induced senescence, ROS, reactive oxygen species, SA-β-gal, senescence-associated β-galactosidase, STAT3, signal transducers and activators of transcription -3, TPA, tissue plasminogen activator

## Abstract

Oncogenic K-Ras (K-Ras^G12V^) promotes senescence in normal cells but fuels transformation of cancer cells after the senescence barrier is bypassed. The mechanisms regulating this pleiotropic function of K-Ras remain to be fully established and bear high pathological significance. We find that K-Ras^G12V^ activates the angiotensinogen (AGT) gene promoter and promotes AGT protein expression in a Kruppel-like factor 6–dependent manner in normal cells. We show that AGT is then converted to angiotensin II (Ang II) in a cell-autonomous manner by cellular proteases. We show that blockade of the Ang II receptor type 1 (AT_1_-R) in normal cells inhibits oncogene-induced senescence. We provide evidence that the oncogenic K-Ras–induced synthesis of Ang II and AT_1_-R activation promote senescence through caveolin-1–dependent and nicotinamide adenine dinucleotide phosphate oxidase 2–mediated oxidative stress. Interestingly, we find that expression of AGT remains elevated in lung cancer cells but in a Kruppel-like factor 6–independent and high-mobility group AT-hook 1–dependent manner. We show that Ang II–mediated activation of the AT_1_-R promotes cell proliferation and anchorage-independent growth of lung cancer cells through a STAT3-dependent pathway. Finally, we find that expression of AGT is elevated in lung tumors of K-Ras^LA2-G12D^ mice, a mouse model of lung cancer, and human lung cancer. Treatment with the AT_1_-R antagonist losartan inhibits lung tumor formation in K-Ras^LA2-G12D^ mice. Together, our data provide evidence of the existence of a novel cell-autonomous and pleiotropic Ang II–dependent signaling pathway through which oncogenic K-Ras promotes oncogene-induced senescence in normal cells while fueling transformation in cancer cells.

WT K-Ras is activated by signals that induce the exchange of bound GDP to GTP. Given the intrinsic ability of K-Ras to hydrolyze GTP, this is a transient activation (1). Single point mutations, including the G12V substitution, generate an oncogenic mutant form of K-Ras with a constitutively activated GTP-bound state. Expression of oncogenic K-Ras promotes oncogene-induced senescence (OIS). Because OIS is an irreversible form of cell-cycle arrest (2, 3), it is a powerful tumor suppressor mechanism. Senescent cells are characterized by enlarged and flat cell morphology. Additional hallmarks of cellular senescence include increased β-galactosidase activity at pH 6, enhanced p53 activity, and elevated p21^Waf1/Cip1^, p16, and γ-H2AX protein expression (4, 5, 6, 7, 8). Senescent cells can also display elevated expression of p19^ARF^, plasminogen activator inhibitor-1 (PAI-1), and DNA damage foci (9) and senescence-associated heterochromatin foci (10) formation. Moreover, senescent cells secrete a plethora of cytokines, growth factors, and proteases, known as the senescence-associated secretory phenotype, which was first identified and functionally characterized by Judith Campisi and her group (11, 12, 13). Consistent with an antitumorigenic role of OIS, senescent cells accumulate at sites of premalignant lesions in mice and humans (7, 14, 15, 16, 17, 18, 19, 20, 21, 22, 23). However, once the senescence barrier is either evaded or bypassed by oncogene-expressing cells, oncogene expression drives cell transformation and tumor growth. Importantly, senescent cells are absent or rare in malignant tumors. The signaling pathways and molecular mechanisms that regulate these pleiotropic roles of oncogenic K-Ras (*i.e.*, antitumorigenic and protumorigenic) remain to be fully understood and are of upmost importance in cancer biology.

The angiotensinogen (AGT)/angiotensin II (Ang II) pathway is a well-established regulator of systemic blood pressure as well as electrolyte and fluid homeostasis. The classical view is that circulating AGT is produced in the liver and is converted to angiotensin I (Ang I) by the kidney-derived renin enzyme. Ang I is then converted to Ang II by the angiotensin-converting enzyme (ACE) on the surface of endothelial cells (24, 25, 26). Ang II acts mostly through activation of the Ang II receptor type 1 (AT_1_-R) (27). Evidence shows that Ang II can be locally formed at much higher concentrations, as compared with systemic Ang II, in the kidney, heart, pancreas, adipose tissue, and vasculature, where AGT, renin, ACE, and AT_1_-R can be locally synthetized (28). In these tissues, Ang II regulates organ homeostasis and the pathogenesis of chronic diseases (28). Nicotinamide adenine dinucleotide phosphate oxidase 2 (NOX2) is the membrane-associated catalytic subunit of NADPH oxidase. NADPH oxidase is a major endogenous source of reactive oxygen species (ROS) (29). Excessive ROS production by NADPH oxidase in vascular cells has been linked to cardiovascular diseases (30). Interestingly, Ang II is also a pathophysiologic stimulus relevant to atherosclerosis and is an activator of NADPH after stimulation of the AT_1_-R (31). However, whether Ang II is locally produced in an autocrine and renin/ACE-independent manner after oncogene activation and the functional significance of local oncogene-induced Ang II signaling are virtually unexplored.

Caveolae are invaginations of the plasma membrane involved in signal transduction (32). Caveolin-1, a structural protein component of caveolae, concentrates and functionally regulates signaling molecules within caveolar membranes. We and others have shown that caveolin-1–mediated signaling plays a key role in linking oxidative stress to cellular senescence. Findings show that the direct regulation of Mdm2 (33), PP2A-C (34), Sirt1 (35), EGF-R (36), Nrf2 (37), TrxR1 (38) and MTH1 (39) by caveolin-1 contributes to the acquisition of a premature senescent phenotype when a cell is hit by a senescent-inducing stimulus, including oxidative stress. A lack of caveolin-1 expression is sufficient to inhibit oxidative stress–induced premature senescence (33, 34, 35, 36, 37, 38, 39).

Lung cancer is the most frequent type of cancer and the most common cause of cancer-related death. Non–small-cell lung cancer (NSCLC) is the most common form of lung cancer, and adenocarcinoma is the most common type of NSCLC (40). Progression from premalignant lesions to malignant adenocarcinomas is a hallmark of NSCLC pathogenesis. In humans, K-Ras mutations represent the most common molecular change in lung adenocarcinomas (41). Signal transducers and activators of transcription-3 (STAT3) is an oncogenic transcription factor. Upon activation, STAT3 translocates to the nucleus where it activates a number of target genes with key roles in proliferation, cell survival, transformation, and chemoresistance (42, 43). STAT3 activity is significantly elevated in NSCLC and increased expression or activation of this pathway portends a poor prognosis in NSCLC and drug resistance (43). The Janus kinases (JAKs) are main activators of STAT3, and both JAK and STAT3 have been investigated as potential targets for cancer treatment.

In the present study, we find that K-Ras^G12V^ transcriptionally activates the AGT gene and promotes AGT protein expression, which is then converted to Ang II in a cell-autonomous and renin/ACE-independent manner. We describe how this K-Ras/AGT/Ang II pathway induces senescence in normal cells while fuels cell transformation in cancer cells through the differential coupling of the AT_1_-R to NOX2 and STAT3 signaling, respectively. Together, these findings provide a novel and clinically significant link between an oncogene and the local production of a biologically active molecule, that is, Ang II.

## Results

### Oncogenic K-Ras upregulates AGT mRNA and protein expression

**Figure 1:**
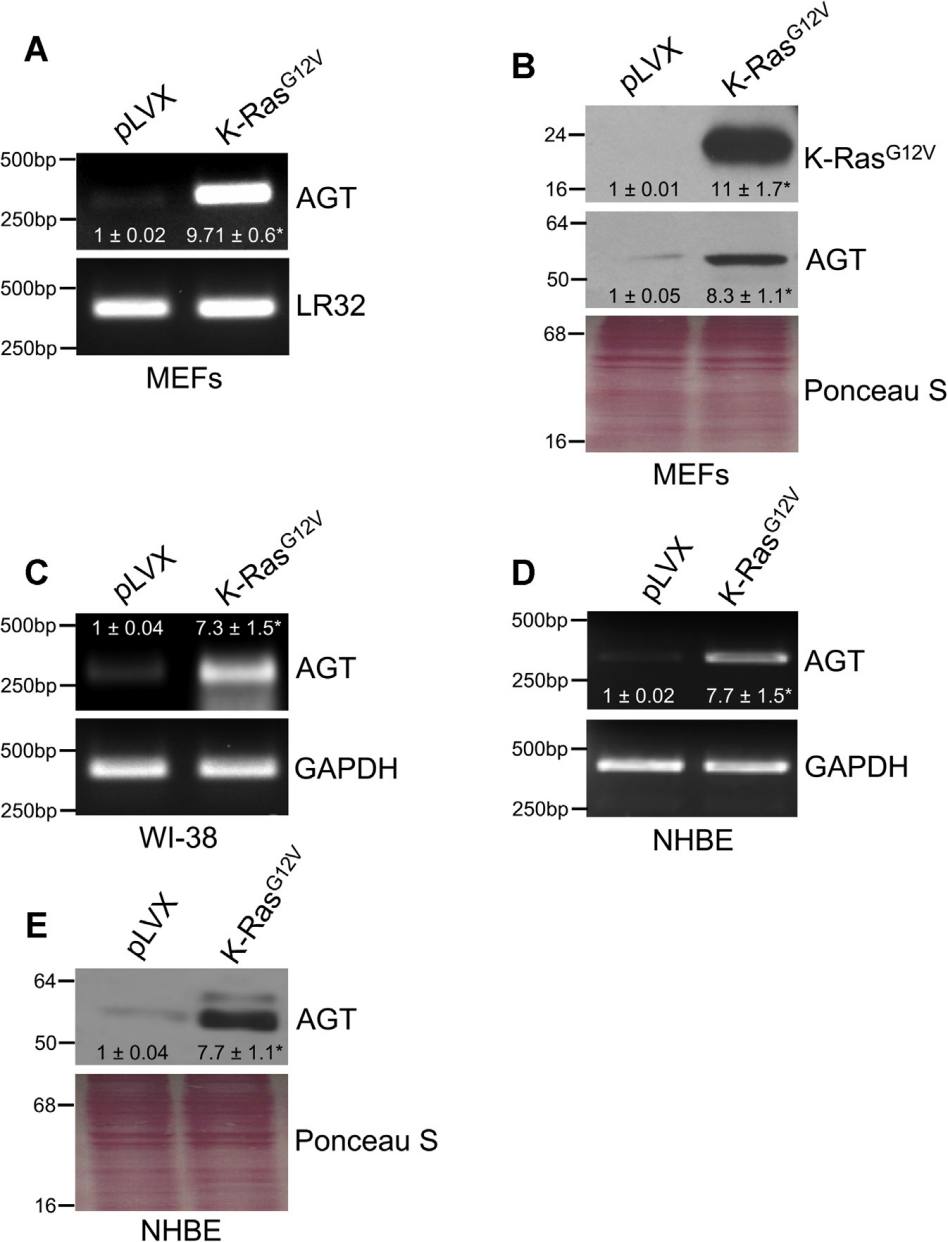
**K-Ras**^**G12V**^**upregulates angiotensinogen expression.***A–B*, mouse embryonic fibroblasts were infected with a lentiviral vector (pLVX) expressing K-Ras^G12V^. Infection of MEFs with pLVX was performed as control. Seven days after infection, angiotensinogen (AGT) mRNA (*A*) and protein (*B*) levels were measured by RT-PCR and immunoblotting analysis, respectively. *C*, angiotensinogen mRNA expression was quantified by RT-PCR analysis in WI-38 human diploid fibroblasts after expression of K-Ras^G12V^. *D–E*, NHBE cells were infected with either pLVX or K-Ras^G12V^. Seven days after infection, AGT mRNA (*D*) and protein (*E*) levels were measured by RT-PCR and immunoblotting analysis, respectively. Expression of LR32 was measured as an internal control in panel *A*. Expression of GAPDH was measured as internal control in panels *C* and *D*. Equal protein loading was assessed by Ponceau S staining in panels *B* and *E*. Blots are representative of three independent experiments. The mean ± SE is shown for each sample. Statistical comparisons were made using Student’s *t*-test. ∗*p* < 0.005. MEF, mouse embryonic fibroblast; NHBE, normal human bronchial epithelial.

To identify novel insights into the molecular signaling mediated by oncogenic K-Ras in normal cells, we tested the overall hypothesis that oncogenic K-Ras regulates the Ang II pathway. The G12V substitution in the K-Ras gene is one of the most common oncogenic mutations found in lung cancer. We began asking whether K-Ras^G12V^ promotes the expression of AGT, the precursor of Ang II. Multiple data show that mouse embryonic fibroblasts (MEFs) are a valid surrogate cellular system in which to model oncogenic K-Ras signaling (44, 45). We find that overexpression of K-Ras^G12V^ in MEFs upregulated both AGT mRNA (Fig. 1*A*) and protein (Fig. 1*B*) expression. Consistent with these data, K-Ras^G12V^ increased AGT mRNA expression in WI-38 human diploid fibroblasts (Fig. 1*C*). Oncogenic K-Ras–mediated upregulation of AGT is not limited to fibroblasts, as we observed a virtually identical outcome in normal human bronchial epithelial (NHBE) cells (Fig. 1, *D* and *E*).

### K-Ras^G12V^ activates the AGT promoter through KLF6

**Figure 2:**
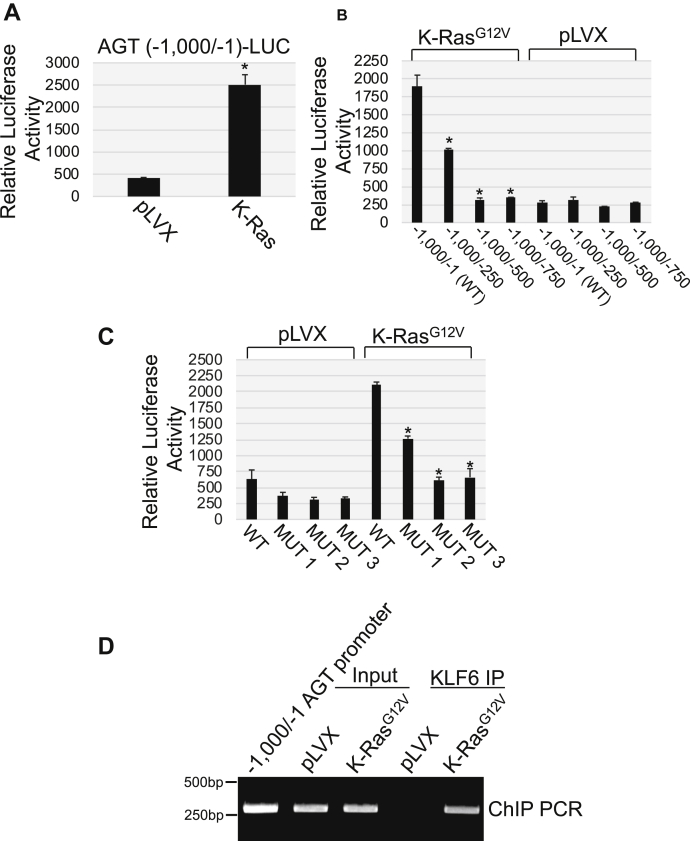
**Oncogenic K-Ras transcriptionally activates the angiotensinogen gene promoter.***A*, mouse embryonic fibroblasts were infected with either vector alone (pLVX) or pLVX-K-Ras^G12V^. After 3 days, cells were transfected with a luciferase-carrying vector (pTA-LUC) under the control of the first 1000 bp of the angiotensinogen gene promoter [AGT (−1000/−1)-LUC]. After 48 h, cells were collected and luciferase activity was measured. *B*, mouse embryonic fibroblasts were infected with either vector alone (pLVX) or pLVX-K-Ras^G12V^. After 3 days, cells were transfected with a luciferase-carrying vector (pTA-LUC) under the control of the first 1000 bp of the angiotensinogen gene promoter (−1000/−1) and the following promoter deletion mutants: (−1000/−250), (−1000/−500), and (−1000/−750). After 48 h, cells were collected and luciferase activity was measured. *C*, mouse embryonic fibroblasts were infected with either vector alone (pLVX) or pLVX-K-Ras^G12V^. After 3 days, cells were transfected with a luciferase-carrying vector (pTA-LUC) under the control of the first 1000 bp of the angiotensinogen gene promoter (−1000/−1) and the following mutants in which each of the three KLF6 binding sites of the AGT promoter was individually disrupted by point mutations to adenine: MUT 1 (KLF site -445), MUT 2 (KLF6 site -354), and MUT 3 (KLF6 site -15). After 48 h, cells were collected and luciferase activity was measured. *D*, MEFs were infected with either pLVX or K-Ras^G12V^. After 5 days, chromatin immunoprecipitation assay was performed using an antibody probe specific for KLF6 and PCR performed using primers flanking the region of the AGT promoter containing KLF6 sites. PCR analysis using input DNA before KLF6 immunoprecipitation was performed to show equal chromatic content. PCR with the first 1000 bp of the angiotensinogen gene promoter (−1000/−1) was performed as positive control. Values in panels *A*, *B,* and *C* represent the means ± SEM. Statistical comparisons were made using Student’s *t*-test. ∗*p* < 0.005. KLF6, Kruppel-like factor 6; AGT, angiotensinogen; MEF, mouse embryonic fibroblast.

Because oncogenic K-Ras upregulated AGT mRNA expression, we asked whether K-Ras^G12V^ transcriptionally activated the AGT gene promoter. We cloned the first 1000 bp of the human AGT promoter upstream of the luciferase gene [AGT (−1000/−1 bp)-LUC]. AGT(−1000/−1 bp)–LUC was expressed in cells in the presence of K-Ras^G12V^ or the empty pLVX vector. We find in Figure 2*A* that oncogenic K-Ras activated the human AGT promoter. Interestingly, deletion mutant assays demonstrate that the region of the AGT promoter ranging from −1 bp to −500 bp was responsive to K-Ras^G12V^ (Fig. 2*B* and Fig. S1*A*). We obtained virtually identical results when the K-Ras^G12V^–induced activation of the AGT promoter was determined in NHBE cells (Fig. S1*B*). *In silico* analysis of the −1 bp/−500 bp region of the AGT promoter showed the existence of potential binding sites for multiple transcription factors (not shown). Kruppel-like factor 6 (KLF6) possesses three putative binding sites at positions −445 bp, −354 bp, and −15 bp from the ATG (Fig. S1*C*). To test the role of KFL6 in the transcriptional regulation of AGT, we generated point mutant forms of the human AGT promoter in which each of the three potential KLF6 binding sites was disrupted (Fig. S1*C*). We find that disruption of each of the three KLF6 binding sites, but particularly the ones at −354 and −15, inhibited oncogenic K-Ras–induced activation of the AGT promoter (Fig. 2*C*). To show direct binding of KLF6 to the KLF6 binding sites of the AGT promoter *in vivo*, we performed chromatin immunoprecipitation (ChIP) analysis on chromatin from pLVX- and K-Ras^G12V^-infected MEFs using an antibody probe specific for KLF6. We show in Figure 2*D* increased binding of KLF6 to the AGT promoter region containing the three KLF6 binding sites after expression of oncogenic K-Ras.

**Figure 3:**
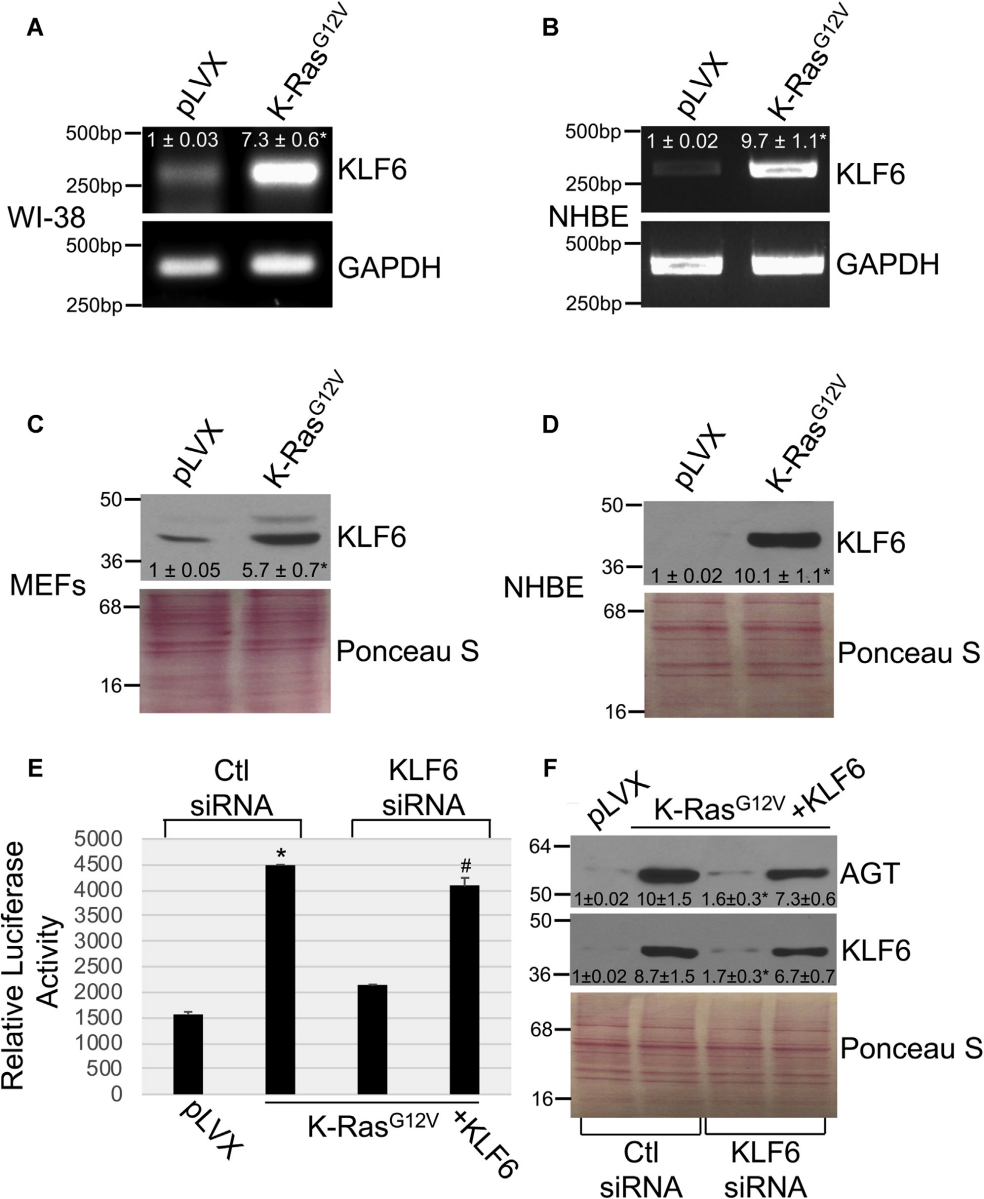
**KLF6 mediates the K-Ras**^**G12V**^**–induced activation of the angiotensinogen promoter.***A–B*, KLF6 mRNA expression was determined by RT-PCR analysis in WI-38 human diploid fibroblasts (*A*) and NHBE cells (*B*) after overexpression of K-Ras^G12V^. Expression of the empty pLVX vector was used as control. RT-PCR using primers specific for GAPDH was performed as an internal control. *C–D*, Cell lysates from mouse embryonic fibroblasts (*C*) and NHBE cells (*D*) were subjected to immunoblotting analysis using an antibody probe specific for KLF6. *E–F*, mouse embryonic fibroblasts were infected with either pLVX or pLVX-K-Ras^G12V^. After 3 days, cells were transfected with either control siRNA (Ctl siRNA) or siRNA against KLF6 (KLF6 siRNA) in the presence or absence of an expression vector carrying the KLF6 cDNA (+KLF6). *E*, after 48 h, cells were transfected with a luciferase-carrying vector (pTA-LUC) under the control of the first 1000 bp of the angiotensinogen gene promoter [AGT (−1000/−1)-LUC]. After 48 h, cells were collected and luciferase activity was measured. *F*, after 48 h, cells were collected and cell lysates were subjected to immunoblotting analysis using anti-KLF6 and anti-AGT IgGs. Values in panel *E* represent the means ± SEM. Equal protein loading was assessed by Ponceau S staining in panels *C, D,* and *F*. Blots are representative of three independent experiments. The mean ± SE is shown for each sample. Statistical comparisons were made using Student’s *t*-test. ∗^,#^*p* < 0.005. NHBE, normal human bronchial epithelial; KLF6, Kruppel-like factor 6; AGT, angiotensinogen; IgG, immunoglobulin G.

These results were corroborated by data showing that K-Ras^G12V^ upregulated KLF6 mRNA (Figs. 3, *A* and *B*) and protein (Fig. 3, *C* and *D*) expression in both fibroblasts and NHBE cells. Importantly, downregulation of KLF6 by siRNA inhibited oncogenic K-Ras–induced activation of the AGT promoter (Fig. 3*E*) and upregulation of AGT protein expression (Fig. 3*F*), which were both rescued when KLF6 was overexpressed in KLF6 siRNA-transfected MEFs (Fig. 3, *E* and *F*). Consistent with these data, we find that downregulation of KLF6 by siRNA inhibited K-Ras^G12V^–mediated upregulation of AGT mRNA in NHBE cells (Fig. S1*D*).

### Oncogenic K-Ras upregulates Ang II levels in a cathepsin D–dependent, chymase-dependent, and TPA-dependent manner

**Figure 4:**
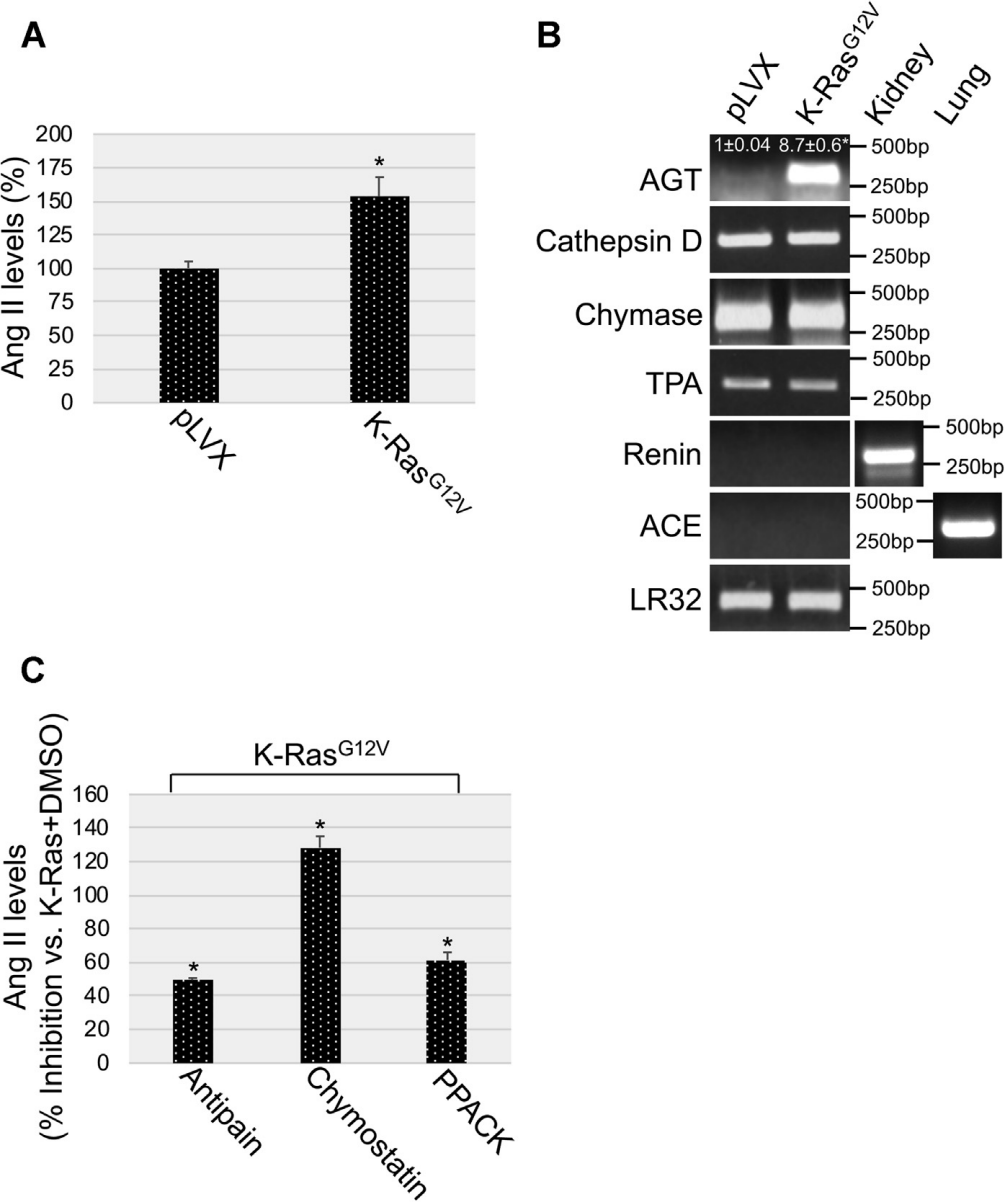
**K-Ras**^**G12V**^**promotes angiotensin II expression in a chymase-dependent, TPA-dependent, and cathepsin D–dependent manner.***A*, mouse embryonic fibroblasts (MEFs) were infected with a lentiviral vector expressing K-Ras^G12V^. Infection with pLVX alone was used as control. After 7 days, angiotensin II (Ang II) was quantified in the conditioned medium derived from pLVX-expressing and pLVX-K-Ras^G12V^–expressing cells. *B*, K-Ras^G12V^ was overexpressed in MEFs as described in panel *A*. After 7 days, expression of angiotensinogen (AGT), chymase, tissue plasminogen activator (TPA), cathepsin D, renin, and angiotensin-converting enzyme (ACE) mRNAs was quantified by RT-PCR analysis. Renin mRNA expression in the kidney and ACE mRNA expression in the lung was determined as control. Quantification of LR32 was performed as an internal control. *C*, K-Ras^G12V^ was overexpressed in MEFs for 7 days in the presence of antipain (5 μM), chymostatin (10 μM), or D-phenylalanyl-L-prolyl-L-arginine chloromethyl ketone (PPACK) (10 μM). Treatment with dimethyl sulfoxide was performed as control. Angiotensin II (Ang II) was quantified in the conditioned medium of these cells. Values in panels *A* and *C* represent the means ± SEM. Statistical comparisons were made using Student’s *t*-test. ∗*p* < 0.005.

AGT is the precursor of Ang II. Because we found in Figures 1 and 2 that oncogenic K-Ras upregulated AGT expression, we asked whether oncogenic K-Ras also elevates Ang II levels. To this end, Ang II levels were quantified by ELISA in cells expressing either K-Ras^G12V^ or the empty pLVX vector. We show in Figure 4*A* that the level of Ang II in the conditioned media of MEFs expressing oncogenic K-Ras was significantly increased, as compared with the media from pLVX-expressing cells. Virtually identical results were obtained when Ang II levels were measured in total cell lysates from K-Ras^G12V^–expressing and pLVX-expressing MEFs (not shown). How is AGT converted to Ang II after expression of oncogenic K-Ras? Renin and ACE, which convert AGT to Ang I and Ang I to Ang II, respectively, are well-established enzymes mediating the classical systemic Ang II synthesis in response to a drop of blood pressure. Interestingly, we did not find expression of either renin or ACE in MEFs (Fig. 4*B*). However, we did find constitutive and K-Ras^G12V^–independent expression of cathepsin D, chymase, and tissue plasminogen activator (TPA), which can mediate the conversion of AGT to Ang I, Ang I to Ang II, and AGT directly to Ang II, respectively (Fig. 4*B*). Importantly, we causally linked these enzymes to Ang II synthesis by showing in Figure 4*C* that treatment with antipain (cathepsin D inhibitor), chymostatin (chymase inhibitor), and D-phenylalanyl-L-prolyl-L-arginine chloromethyl ketone (PPACK) (TPA inhibitor) significantly inhibited oncogenic K-Ras–induced synthesis of Ang II in MEFs. We obtained virtually identical results in NHBE cells (Fig. S2, *A–D*). Together, our data show that oncogenic K-Ras upregulates AGT protein levels by transcriptionally activating the AGT gene promoter and that AGT is then converted to Ang II by cathepsin D, chymase, and TPA in a cell-autonomous and renin/ACE-independent manner.

### AT_1_-R antagonists inhibit K-Ras^G12V^–mediated cellular senescence

**Figure 5:**
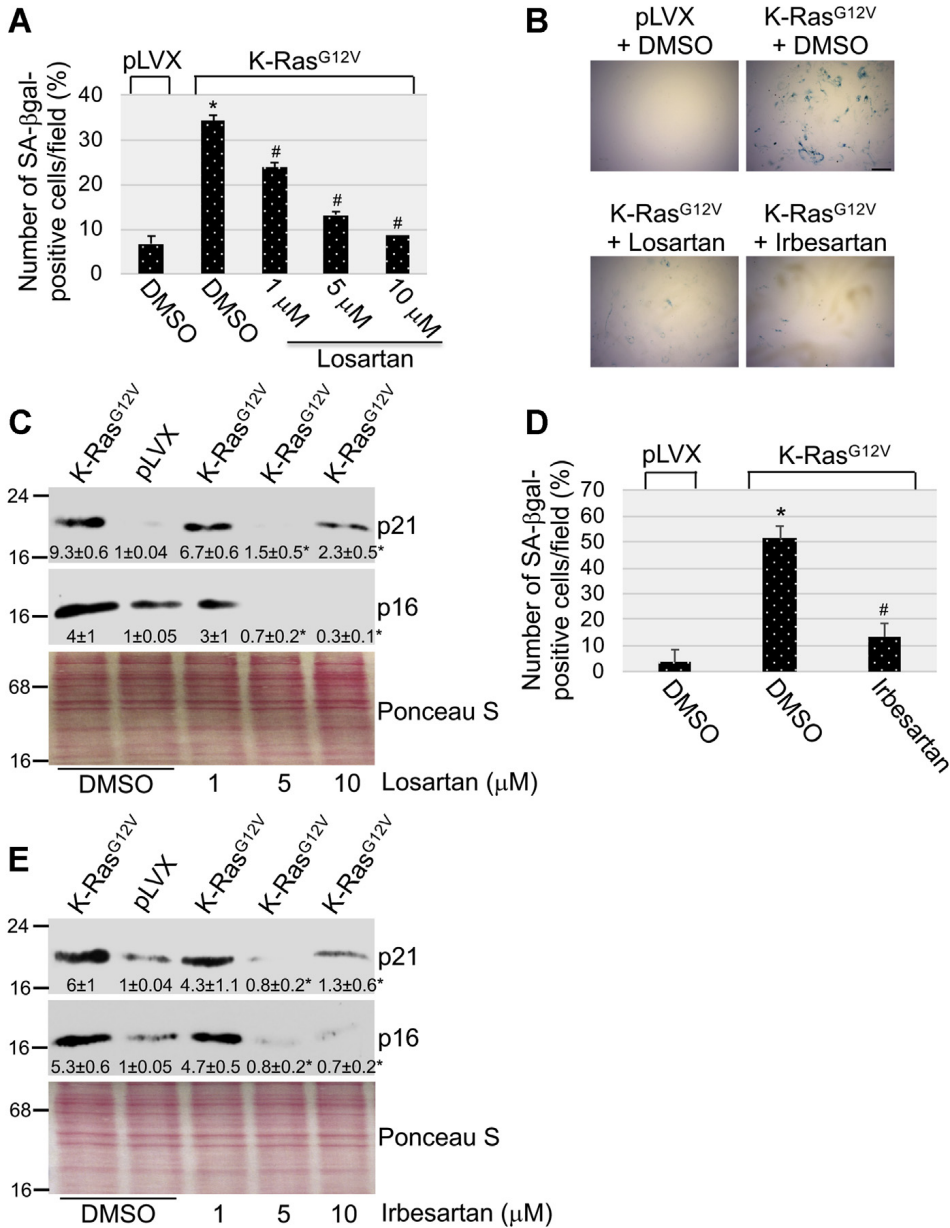
**AT**_**1**_**-R antagonists inhibit oncogene-induced senescence.***A–C*, mouse embryonic fibroblasts were infected with a lentiviral vector (pLVX) expressing K-Ras^G12V^. Infection of MEFs with pLVX was performed as control. Cells were cultured for 10 days in the presence of different concentrations of losartan. Treatment with dimethyl sulfoxide served as control. In panels *A* and *B*, cells were subjected to senescence-associated β-galactosidase (SA-β-gal) staining. Quantification is shown in panel *A*, representative images are shown in panel *B*. Scale bar = 100 μm. In panel *C*, cell lysates were subjected to immunoblotting analysis using antibody probes specific for p21 and p16. *D* and *E*, mouse embryonic fibroblasts were infected with a lentiviral vector (pLVX) expressing K-Ras^G12V^. Infection of MEFs with pLVX was performed as control. Cells were cultured for 10 days in the presence of different concentrations of irbesartan. Treatment with dimethyl sulfoxide served as control. Cells were subjected to senescence-associated β-galactosidase (SA-β-gal) staining. Quantification is shown in panel *D*. In panel *E*, cell lysates were subjected to immunoblotting analysis using antibody probes specific for p21 and p16. Values in panels *A* and *D* represent the means ± SEM. Equal protein loading was assessed by Ponceau S staining in panels *C* and *E*. Blots are representative of three independent experiments. The mean ± SE is shown for each sample. Statistical comparisons were made using Student’s *t*-test. ∗^,#^*p* < 0.005. MEF, mouse embryonic fibroblast; AT_1_-R, Ang II receptor type 1.

The AT_1_-R is the main receptor through which Ang II transduces its action. Overexpression of oncogenic K-Ras is known to promote premature senescence in normal cells. To directly investigate the functional significance of oncogenic K-Ras–mediated upregulation of Ang II expression, we asked whether K-Ras^G12V^ promotes cellular senescence in an Ang II-dependent manner in normal cells. To this end, K-Ras^G12V^ was expressed in MEFs in which the AT_1_-R was inhibited by losartan, a highly selective AT_1_-R antagonist. Expression of the empty pLVX vector and treatment with dimethyl sulfoxide (DMSO) were used as controls. We find that treatment with losartan inhibited the K-Ras^G12V^–induced upregulation of the senescence markers senescence-associated β-galactosidase (SA-β-gal) activity (Figs. 5, *A* and *B*), and p16 and p21 protein expression (Fig. 5*C*) in MEFs. These findings were corroborated by the ability of Irbesartan, another selective AT_1_-R antagonist, to inhibit K-Ras^G12V^–induced increase of SA-β-gal activity (Fig. 5, *B* and *D*), and p16 and p21 protein expression (Fig. 5*E*). Consistent with these findings, we find that oncogenic K-Ras–induced premature senescence was inhibited in NHBE cells when either the AT_1_-R was inhibited by the AT_1_-R–specific antagonist losartan or Ang II synthesis was inhibited by treatment with chymostatin (Fig. S3, *A–D*). Thus, Ang II–mediated activation of the AT_1_-R is promoted by oncogenic K-Ras and mediates OIS.

### Oncogenic K-Ras promotes cellular senescence in an AT_1_-R/NOX2/ROS-dependent manner

**Figure 6:**
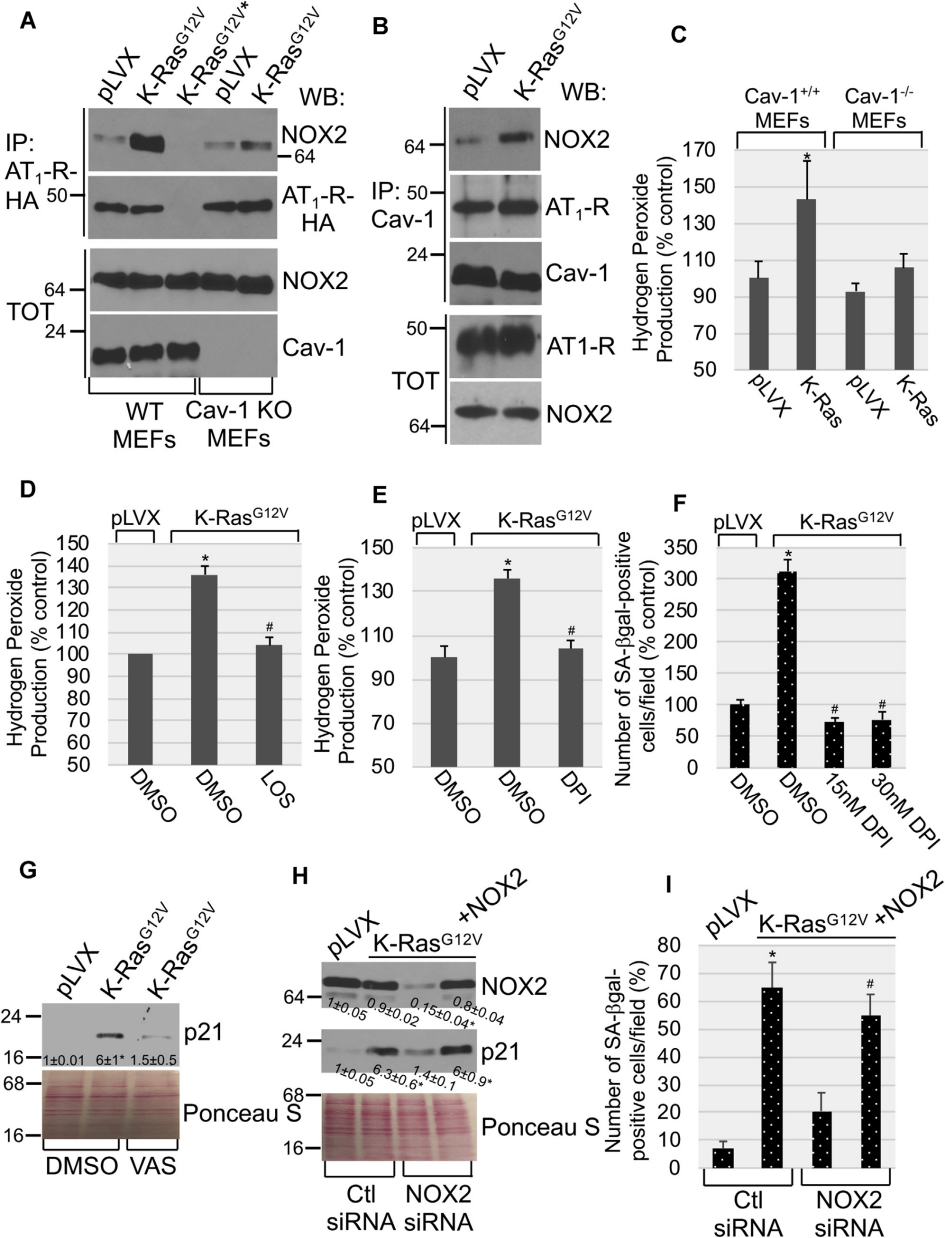
**K-Ras**^**G12V**^**induces cellular senescence in a AT**_**1**_**-R/NOX2-dependent manner.***A*, WT and caveolin-1 KO MEFs were infected with pLVX or pLVX-K-Ras^G12V^ and cultured for 5 days. AT_1_-R-HA and NOX2 were then transiently transfected in pLVX-expressing and pLVX-K-Ras^G12V^–expressing MEFs. After 2 days, cell lysates were immunoprecipitated with an antibody specific for HA-tag, and immunoprecipitates were subjected to immunoblotting analysis with anti-NOX2 and anti-HA IgGs. Total expression of NOX2 and caveolin-1 is shown in the lower panels. An ∗ is used to indicate transfection of NOX2 but not AT_1_-R-HA in WT MEFs, which was performed as an internal control. *B*, WT MEFs were infected with pLVX or pLVX-K-Ras^G12V^ and cultured for 5 days. AT_1_-R-HA and NOX2 were then transiently transfected in pLVX--expressing and pLVX-K-Ras^G12V^–expressing MEFs. After 2 days, cell lysates were immunoprecipitated with an antibody specific for caveolin-1, and immunoprecipitates were subjected to immunoblotting analysis with anti-HA tag, anti-NOX2, and anti–caveolin-1 IgGs. Total expression of AT_1_-R-HA and NOX2 is shown in the lower panels. *C*, WT and caveolin-1 null MEFs were infected with pLVX or pLVX-K-Ras^G12V^ and cultured for 7 days. Hydrogen peroxide levels were measured by Amplex Red assay. *D*, WT MEFs were infected with pLVX or pLVX-K-Ras^G12V^ and cultured for 10 days in the presence of losartan (5 μM). Treatment with dimethyl sulfoxide (DMSO) was performed as control. Hydrogen peroxide levels were measured by Amplex Red assay. *E–F*, MEFs were infected with pLVX or pLVX-K-Ras^G12V^ and cultured for 10 days in the presence of either DMSO or DPI. Hydrogen peroxide levels were measured by Amplex Red assay (*E*), and senescence was quantified by SA-β-gal staining (*F*). *G*, WT MEFs were infected with pLVX or pLVX-K-Ras^G12V^ and cultured for 10 days in the presence of VAS2870 (4 μM). Treatment with DMSO was performed as control. p21 expression was determined by immunoblotting using a p21-specific antibody probe. *H* and *I,* K-Ras^G12V^ was expressed in WT mouse embryonic fibroblasts. Infection of MEFs with the empty pLVX vector served as control. After 3 days, cells were transfected with either control siRNA (Ctl siRNA) or NOX2 siRNA in the presence or absence of an expression vector carrying the NOX2 cDNA (+NOX2). Cells were cultured for 7 additional days. In panel *H*, p21 and NOX2 protein expression was assessed by immunoblotting analysis with anti-p21 and anti-NOX2 IgGs, respectively. In panel *I*, cellular senescence was quantified by SA-β-gal staining. Values in panels *C–F* and *I* represent the means ± SEM. Equal protein loading was assessed by Ponceau S staining in panels *G* and *H*. Blots are representative of three independent experiments. The mean ± SE is shown for each sample. Statistical comparisons were made using Student’s *t*-test. ∗*p* < 0.005. IgG, immunoglobulin G; NOX2, nicotinamide adenine dinucleotide phosphate oxidase 2; MEF, mouse embryonic fibroblast; AT_1_-R, Ang II receptor type 1.

What is the signaling, downstream of the Ang II-AT_1_-R, that drives oncogenic K-Ras–mediated cellular senescence? NADPH oxidase (NOX) is the major source of hydrogen peroxide in mammalian cells. Although oncogenic K-Ras is known to promote OIS in an ROS-dependent manner, the underlying molecular mechanisms remain to be fully established. Because our data indicate that K-Ras^G12V^ promoted OIS in an AT_1_-R–dependent manner, we tested the hypothesis that oncogenic K-Ras couples the AT_1_-R, after activation by Ang II, to NOX2. Our coimmunoprecipitation studies show that expression of K-Ras^G12V^ promoted the interaction between the AT_1_-R and NOX2 (Fig. 6*A*). Caveolae are invaginations of the plasma membranes involved in signal transduction. Caveolin-1 is a structural protein component of caveolae that concentrates and functionally regulates signaling molecules within caveolar membranes. Interestingly, we find that the AT_1_-R interacted with caveolin-1 both under resting conditions and after oncogenic K-Ras expression (Fig. 6*B*). In contrast, the interaction of NOX2 with caveolin-1 was promoted by K-Ras^G12V^ (Fig. 6*B*), suggesting that oncogenic K-Ras stimulated the recruitment of NOX2 to caveolar membranes. In support of this conclusion, we find that oncogenic K-Ras failed to promote the interaction of the AT_1_-R with NOX2 in caveolin-1 null MEFs (Fig. 6*A*). NOX2 localization in caveolae was necessary for NOX2 activity, as shown by the inability of K-Ras^G12V^ to induce hydrogen peroxide synthesis in cells lacking caveolin-1 expression (Fig. 6*C*). Activation of the AT_1_-R was also necessary for NOX activity, as shown by the ability of losartan to significantly inhibit K-Ras^G12V^–induced hydrogen peroxide production (Fig. 6*D*). In support of these data, downregulation of the AT_1_-R by siRNA (Fig. S4*A*) also inhibited hydrogen peroxide production induced by the expression of K-Ras^G12V^ (Fig. S4*B*).

To determine the functional consequence of ROS production induced by the oncogenic K-Ras–initiated and Ang II/AT_1_-R-mediated activation of NOX2, we first quantified cellular senescence in cells in which ROS production was inhibited by treatment with diphenyleneiodonium chloride (DPI), an inhibitor of ROS production. We find that ablation of K-Ras^G12V^–induced ROS production by DPI (Fig. 6*E*) prevented oncogenic K-Ras–mediated premature senescence (Fig. 6*F*). To directly link this observed outcome to NOX2 function, we show that treatment with VAS2870, a NOX inhibitor, significantly prevented the ability of K-Ras^G12V^ to upregulate the senescence marker p21 (Fig. 6*G*). Consistent with these findings, downregulation of NOX2 expression by siRNA (Fig. 6*H*) inhibited the K-Ras^G12V^–induced upregulation of p21 expression (Fig. 6*H*) and accumulation of SA-β-gal–positive cells (Fig. 6*I*). Importantly, when we rescued NOX2 expression in NOX2 siRNA-transfected cells (Fig. 6*H*), we also rescued the ability of oncogenic K-Ras to promote premature senescence (Fig. 6, *H* and *I*).

Together, these data suggest that after oncogenic K-Ras expression, the AGT promoter is activated and Ang II expression is upregulated, which leads to activation of the AT_1_-R. Activation of the AT_1_-R leads to activation of NOX2 in caveolae, which triggers NOX2-mediated ROS production and ROS-dependent premature senescence.

### HMGA1 upregulates AGT expression in lung cancer cells

**Figure 7:**
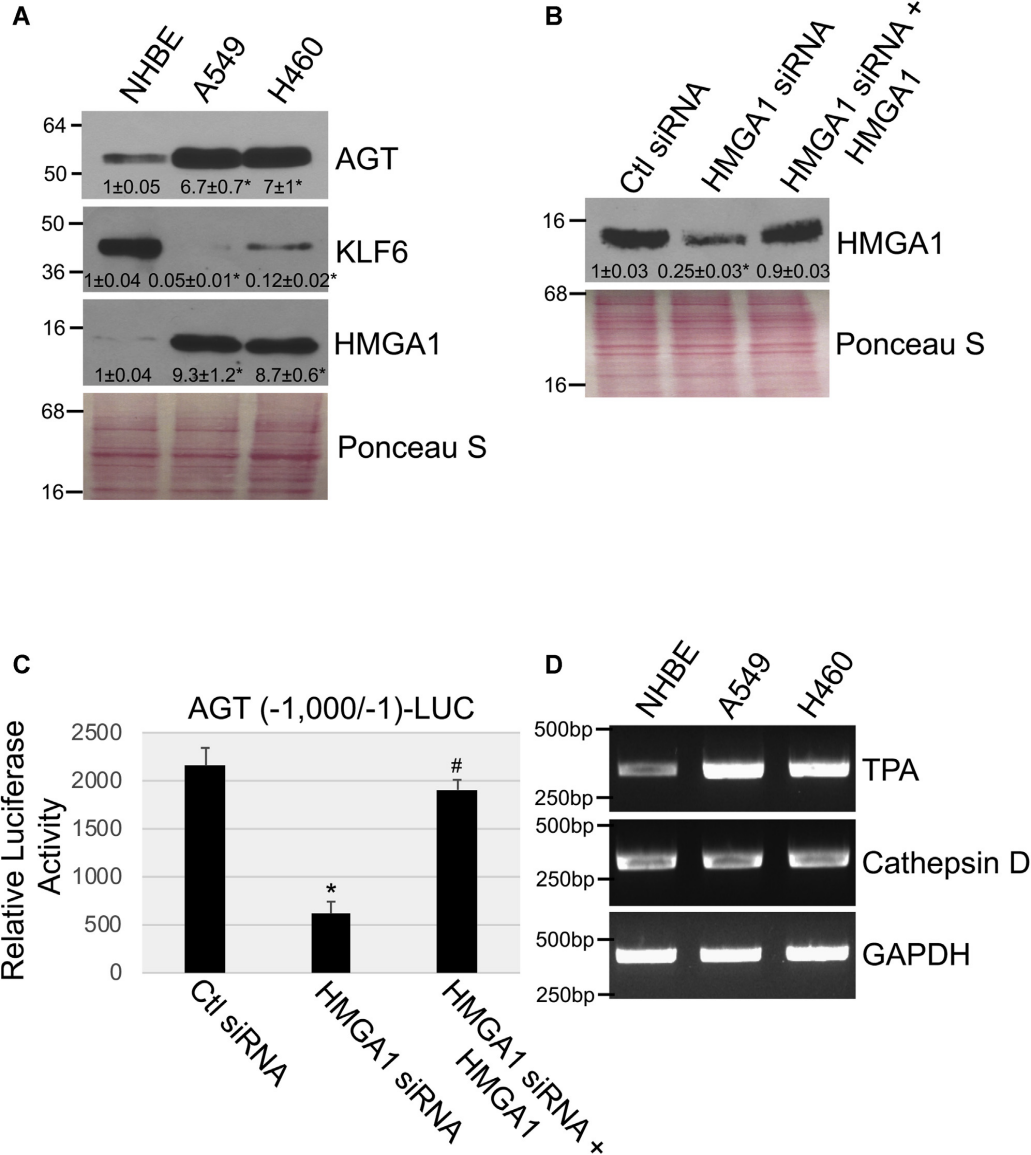
**HMGA1 mediates angiotensinogen expression in NSCLC cells.***A*, angiotensinogen (AGT), KLF6, and HMGA1 protein expression in NHBE, A549, and H460 cells was determined by immunoblotting analysis. *B* and *C*, A549 NSCLC cells were transfected with either control siRNA (Ctl siRNA) or HMGA1 siRNA in the presence or absence of an expression vector carrying the HMGA1 cDNA (+HMGA1). In panel *B*, after 96 h, cells were collected and subjected to immunoblotting analysis using an antibody probe specific for HMGA1. In panel *C*, after 48 h, cells were transfected with a luciferase-carrying vector (pTA-LUC) under the control of the first 1000 bp of the angiotensinogen gene promoter [AGT (−1000/−1)-LUC]. After 48 h, cells were collected and luciferase activity was measured. *D*, TPA and cathepsin D mRNA levels were determined by RT-PCR in NHBE, A549, and H460 cells. Amplification of GAPDH served as internal control. Values in panel *C* represent the means ± SEM. Equal protein loading was assessed by Ponceau S staining in panels *A* and *B*. Blots are representative of three independent experiments. The mean ± SE is shown for each sample. Statistical comparisons were made using Student’s *t*-test. ∗^,#^*p* < 0.005. NHBE, normal human bronchial epithelial; HMGA1, high-mobility group AT-hook 1; KLF6, Kruppel-like factor 6; TPA, tissue plasminogen activator; NSCLC, non–small-cell lung cancer.

Because cellular senescence is a tumor suppressor mechanism and our data show that K-Ras^G12V^ promotes premature senescence through upregulation of the AGT/Ang II pathway, we then asked whether activation of this signaling pathway was inhibited in lung cancer cells carrying oncogenic K-Ras, which have bypassed senescence and possess a transformed phenotype. For these studies, we used A549 and H460 NSCLC cells, well-established cellular models of lung cancer carrying oncogenic K-Ras mutations at residues G12 (A549) and Q61 (H460), which are commonly found in human lung cancer. To our surprise, we find that AGT expression was elevated in oncogenic K-Ras–carrying A549 and H460 cells, as compared with NHBE cells, which do not express oncogenic K-Ras (Fig. 7*A*). In contrast to what we found in normal cells after overexpression of K-Ras^G12V^ (Fig. 3), AGT expression was not mediated by the transcription factor KLF6 in lung cancer cells, in which KLF6 expression was dramatically downregulated (Fig. 7*A*). Interestingly, the AGT promoter has two putative binding sites for the transcription factor high-mobility group AT-hook 1 (HMGA1) at positions −996 and −651 (Fig. S5*A*) and we find in Fig. 7*A* that HMGA1 expression was elevated in both A549 and H460 lung cancer cells. Downregulation of HMGA1 by siRNA (Fig. 7*B*) inhibited AGT promoter activity in A549 lung cancer cells (Fig. 7*C*). When we overexpressed HMGA1 in HMGA1 siRNA-transfected lung cancer cells to restore HMGA1 expression (Fig. 7*B*), we rescued AGT promoter activity in these cells (Fig. 7*C*). We conclude that although KLF6 promotes AGT gene transcription when oncogenic K-Ras is expressed in normal cells, HMGA1 promotes AGT expression in lung cancer cells expressing oncogenic K-Ras. Interestingly, cathepsin D and TPA (Fig. 7*D*), but not chymase, renin and ACE (not shown), were expressed in both A549 and H460 lung cancer cells and TPA levels were upregulated in NSCLC cells, as compared with NHBE cells (Fig. 7*D*). These data suggest that AGT, similarly to what happens in normal cells (Fig. 4), may be converted to Ang II in a TPA-dependent and cathepsin D–dependent but renin-/ACE-independent manner also in NSCLC cells.

### Inhibition of the AGT/Ang II/AT_1_-R signaling inhibits proliferation and transformation properties of lung cancer cells

**Figure 8:**
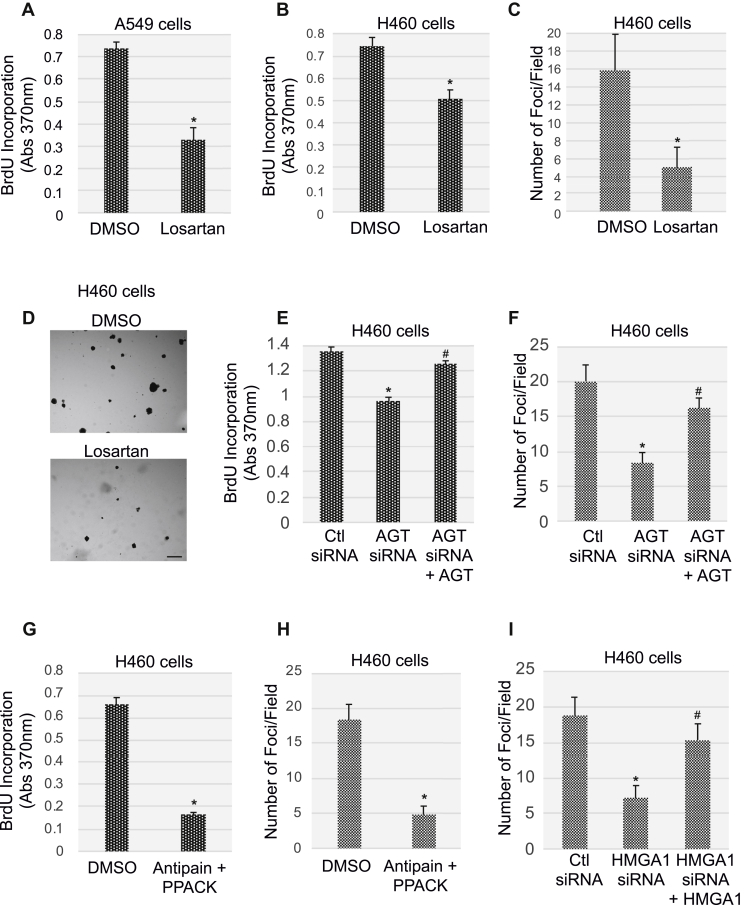
**Inhibition of AT**_**1**_**-R activation, angiotensinogen expression, and angiotensin II synthesis suppresses NSCLC proliferation and transformation.***A–B*, A549 (*A*) and H460 (*B*) cells were treated with 5-μM losartan for 48 h. Treatment with dimethyl sulfoxide (DMSO) served as control. Cell proliferation was then quantified by BrdU incorporation assay. *C–D*, H460 cells were treated with either DMSO or 5-μM losartan for 48 h. Cells were then collected, and 5 x 10^4^ cells were cultured in soft agar for 7 days in the presence of either DMSO or losartan. Quantification of colony formation is shown in panel *C*, representative images are shown in panel *D*. Scale bar = 500 μm. *E* and *F* H460 cells were transfected with either control siRNA (Ctl siRNA) or angiotensinogen (AGT) siRNA in the presence or absence of an expression vector carrying the AGT cDNA (+AGT). After 72 h, cellular proliferation was quantified by BrdU incorporation assay (*E*) while cell transformation was quantified by growth in soft agar for 7 days (*F*). *G* and *H* H460 cells were treated with antipain (5 μM) and D-phenylalanyl-L-prolyl-L-arginine chloromethyl ketone (PPACK) (10 μM) for 72 h. Treatment with DMSO was performed as control. Cellular proliferation was quantified by BrdU incorporation assay (*G*), whereas cell transformation was quantified by growth in soft agar for 7 days in the presence of either DMSO or antipain and PPACK (*H*). *I*, H460 NSCLC cells were transfected with either control siRNA (Ctl siRNA) or HMGA1 siRNA in the presence or absence of an expression vector carrying the HMGA1 cDNA (+HMGA1). After 72 h, cells were collected and subjected to growth in soft agar assay for 7 days. Values in panels *A–C* and *E–I* represent the means ± SEM. Statistical comparisons were made using Student’s *t*-test. ∗^,#^*p* < 0.005. NSCLC, non–small-cell lung cancer; HMGA1, high-mobility group AT-hook 1; AGT, angiotensinogen; AT_1_-R, Ang II receptor type 1.

What is the functional role of the AGT/Ang II/AT_1_-R pathway in lung cancer cells? To directly answer this question, we first inhibited activation of the AT_1_-R with the antagonist losartan. We find that AT_1_-R blockade inhibited cellular proliferation and anchorage-independent growth of lung cancer cells, as quantified by BrdU incorporation (Fig. 8, *A* and *B*) and growth in soft agar (Fig. 8, *C* and *D*) assays, respectively. Consistent with these data, we show in Figure 8, *E* and *F* that downregulation of AGT expression by siRNA (Fig. S5*B*) inhibited cellular proliferation and growth in soft agar of lung cancer cells, respectively. Importantly, rescue of AGT expression in AGT siRNA-expressing H460 cells (Fig. S5*B*) rescued the ability of these cells to incorporate BrdU (Fig. 8*E*) and form colonies in soft agar (Fig. 8*F*). Similarly, cellular proliferation (Fig. 8*G*) and growth in soft agar (Fig. 8*H*) were significantly compromised when lung cancer cells were cotreated with the Ang II synthesis inhibitors antipain and PPACK, which we show inhibit cathepsin D (Fig. S5*C*) and TPA (Fig. S5*D*) activity, respectively. Finally, downregulation by siRNA of HMGA1 (Fig. S5*E*), a positive regulator of AGT transcription in lung cancer cells (Fig. 7), inhibited growth in soft agar of H460 cells (Fig. 8*I*). Restoration of HMGA1 expression in HMGA1 siRNA-expressing H460 cells (Fig. S5*E*) rescued the colony formation properties of H460 cells (Fig. 8*I*). Thus, the cell autonomous AGT/Ang II/AT_1_-R pathway possesses protumorigenic properties in NSCLC cells.

### Activation of STAT3 by the AT_1_-R drives the transformed phenotype of lung cancer cells

**Figure 9:**
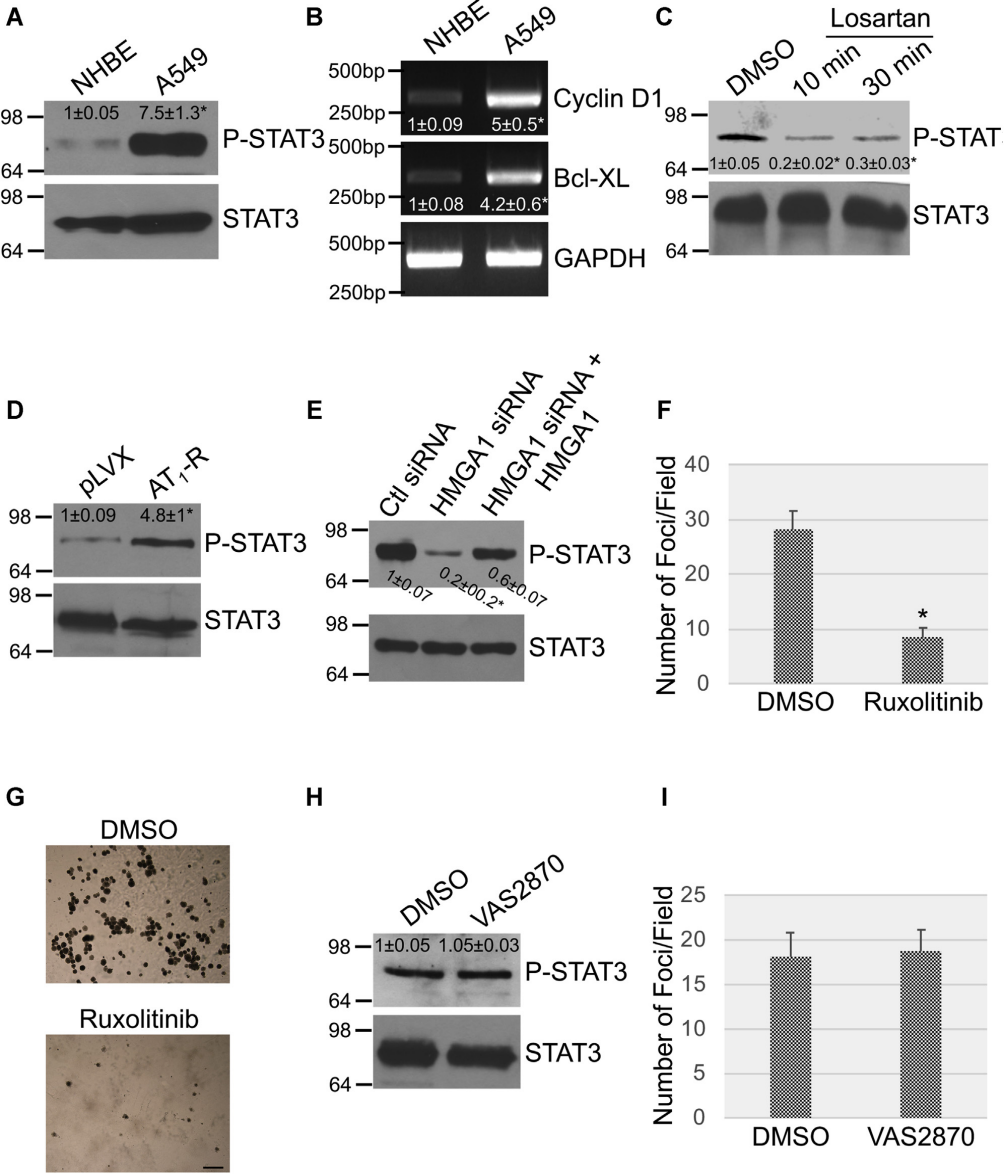
**Activation of STAT3 by the angiotensinogen/AT**_**1**_**-R pathway promotes the transformed phenotype of NSCLC cells.***A*, cell lysates from NHBE and A549 cells were subjected to immunoblotting analysis using an antibody probe specific for phosphorylated (Tyr705) STAT3. Immunoblotting using total STAT3 IgGs served as control. *B*, expression of cyclin D1 and Bcl-XL mRNA in NHBE and A549 cells was determined by RT-PCR analysis. RT-PCR using GAPDH-specific primers was performed as internal control. *C*, A549 cells were treated with 5-μM losartan for either 10 or 30 min. Treatment with dimethyl sulfoxide (DMSO) served as control. Expression of phospho(Tyr705) STAT3 and total STAT3 was determined by immunoblotting analysis. *D*, A549 cells were infected with a lentiviral vector overexpressing AT_1_-R. Expression of the pLVX vector alone was used as control. After 72 h, cells were collected and phospho(Tyr705) STAT3 expression was determined by immunoblotting analysis. Total STAT3 expression was determined as internal control. *E*, A549 cells were transfected with control siRNA (Ctl siRNA) or HMGA1 siRNA in the presence or absence of an expression vector carrying the HMGA1 cDNA (+HMGA1). After 72 h, cell lysates were subjected to immunoblotting analysis using antibody probes specific for phospho(Tyr705) STAT3 and total STAT3. Expression of HMGA1 was quantified as in Figure 7*B*. *F* and *G*, H460 cells were treated with either DMSO or ruxolitinib (5 μM) for 72 h. Cells were then collected, and 5 x 10^4^ cells were cultured in soft agar for 7 days in the presence of either DMSO or ruxolitinib. Quantification of colony formation is shown in panel *F*, representative images are shown in panel *G*. Scale bar = 500 μm. *H* and *I* H460 cells were treated with either DMSO or VAS2870 (4 μM) for 72 h. Cell lysates were subjected to immunoblotting analysis with anti-phospho(Tyr705)-STAT3 IgGs and anti-total STAT3 IgGs (*H*). Cells were also collected and 5 x 10^4^ cells were cultured in soft agar for 7 days in the presence of either DMSO or ruxolitinib. Quantification of colony formation is shown in panel *I*. Values in panels *F* and *I* represent the means ± SEM. Statistical comparisons were made using Student’s *t*-test. ∗*p* < 0.005. IgG, immunoglobulin G; HMGA1, high-mobility group AT-hook 1; STAT3, signal transducers and activators of transcription-3; NHBE, normal human bronchial epithelial; AT_1_-R, Ang II receptor type 1; NSCLC, non–small-cell lung cancer.

Translocation of the transcription factor STAT3 to the nucleus results in the activation of a number of target genes with key roles in proliferation, cell survival, transformation, and chemoresistance (42, 43). STAT3 activity is significantly elevated in NSCLC cells and increased expression or activation of this pathway portends a poor prognosis in NSCLC and drug resistance (43). Here, we tested the hypothesis that activation of STAT3 is the signaling pathway, downstream of the AGT/Ang II-mediated activation of the AT_1_-R, that fuels the transformed phenotype of lung cancer cells. We demonstrate that STAT3 was indeed activated in NSCLC cells, as shown by increased expression of phospho(Tyr705)-STAT3 by immunoblotting (Fig. 9*A*) and of cyclin D1 and Bcl-XL, STAT3 targets, by RT-PCR (Fig. 9*B*) analysis, as compared with NHBE cells. Interestingly, blockade of the AT_1_-R with losartan inhibited the expression of phospho(Tyr705)-STAT3 (Fig. 9*C*) in lung cancer cells, suggesting that STAT3 activation is, at least in part, dependent upon activation of the AT_1_-R. Consistent with these data, overexpression of the AT_1_-R activated STAT3 (Fig. 9*D*), whereas downregulation of HMGA1 by siRNA (Fig. 7*B*) inhibited STAT3 in A549 cells (Fig. 9*E*). Rescue of HMGA1 expression in HMGA1 siRNA-expressing A549 cells (Fig. 7*B*) restored STAT3 activation in these cells (Fig. 9*E*). Virtually identical results were obtained in H460 cells (not shown). Importantly, we show in Figure 9, F and G that inhibition of JAK, an upstream activator of STAT3, with Ruxolitinib inhibited the growth in soft agar of lung cancer cells. Finally, we find that inhibition of NOX with VAS2870 did not inhibit STAT3 (Fig. 9*H*) in lung cancer cells and did not affect their transformed phenotype (Fig. 9*I*), suggesting that the transformation properties of oncogenic K-Ras–expressing lung cancer cells rely upon coupling of the AT_1_-R with STAT3 but not NOX.

### AGT is upregulated in human lung cancer, and losartan inhibits tumorigenesis in a mouse model of lung cancer

**Figure 10:**
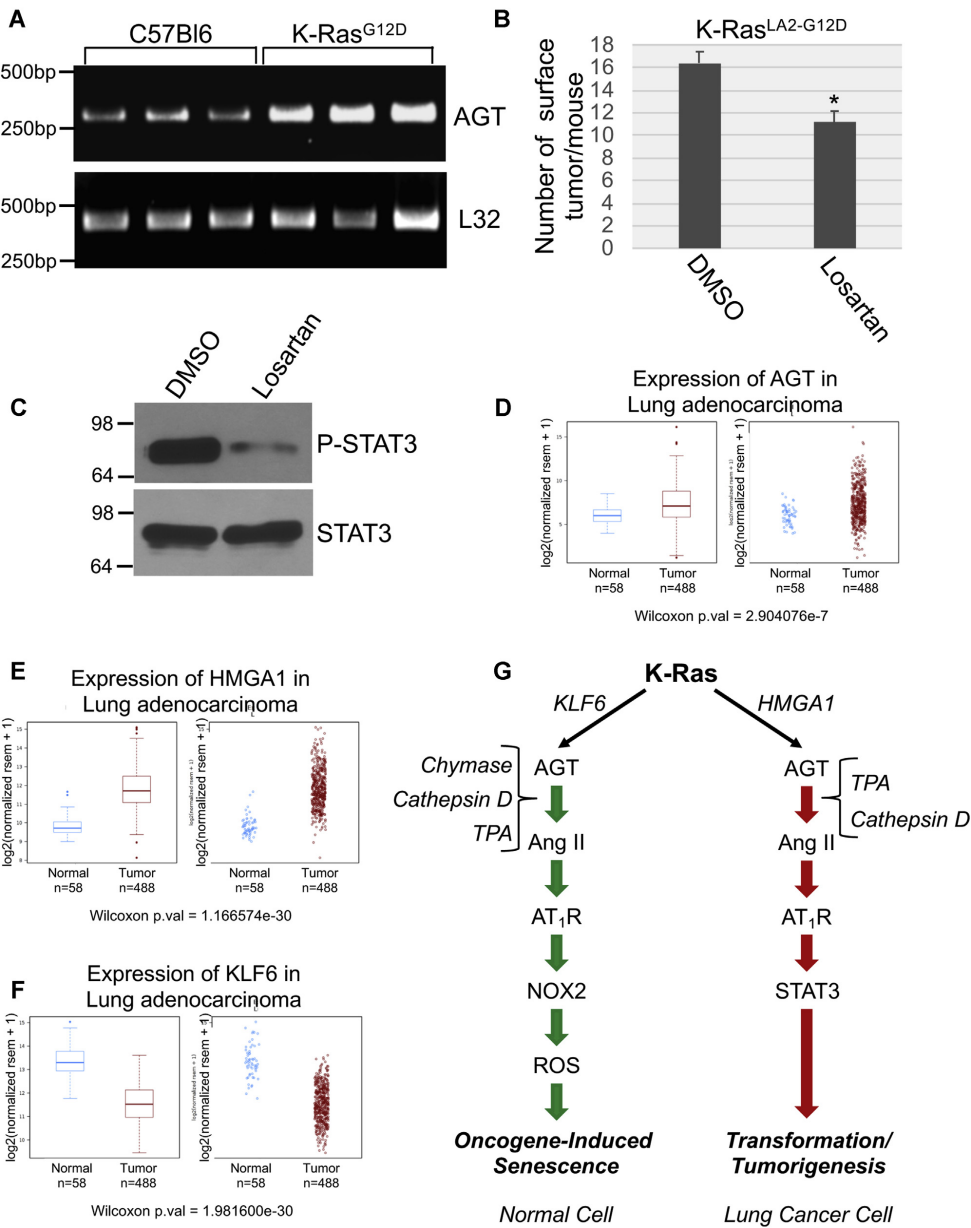
**Angiotensinogen expression is upregulated in the lungs of K-Ras**^**G12D**^**mice and human lung adenocarcinoma. Losartan inhibits lung tumor formation in K-Ras**^**G12D**^**mice.***A*, angiotensinogen mRNA expression in the lungs of C57Bl6 and K-Ras^G12D^ mice was determined by RT-PCR analysis using primers specific for angiotensinogen. Expression of L32 mRNA served as an internal control. *B–C*, K-Ras^G12D^ mice (2 weeks old) were subjected to daily intraperitoneal injections with 50 mg/kg losartan (n = 7). Injection with dimethyl sulfoxide was performed as control (n = 5). After 14 days, mice were sacrificed. In panel *B*, the number of surface lung tumors was counted. In panel *C*, lung lysates consisting of equal total proteins from three dimethyl sulfoxide- and three losartan-injected mice were subjected to immunoblotting analysis using anti-phospho(Tyr705)-STAT3 IgGs and anti-total STAT3 IgGs. *D* and *F*, Gene expression analysis of angiotensinogen (*D*), HMGA1 (*E*), and KLF6 (*F*) in normal lung and in the lung of adenocarcinoma patients using The Cancer Genome Atlas database. *G*, the schematic diagram summarizing the cell autonomous angiotensinogen–angiotensin II signaling that controls the pleiotropic functions of oncogenic K-Ras. In normal cells, the transcription factor KLF6 mediates the oncogenic K-Ras–dependent activation of the angiotensinogen (AGT) gene promoter. AGT is converted to angiotensin II (Ang II) in a cell-autonomous fashion in a chymase-dependent, cathepsin D–dependent, and TPA-dependent manner. Activation of the AT_1_-R by Ang II in normal cells promotes cellular senescence through the NOX2-dependent generation of reactive oxygen species (ROS). In NSCLC cells, the transcription factor HMGA1 mediates the oncogenic K-Ras–dependent activation of the angiotensinogen gene promoter. AGT is converted to angiotensin II (Ang II) in a cell-autonomous fashion in a TPA-dependent and cathepsin D–dependent manner. Activation of the AT_1_-R by Ang II in NSCLC cells promotes proliferation and cell transformation through activation of STAT3. Values in panel *B* represent the means ± SEM. Statistical comparisons were made using Student’s *t*-test. ∗*p* < 0.005. TPA, tissue plasminogen activator; NSCLC, non–small-cell lung cancer; IgG, immunoglobulin G; HMGA1, high-mobility group AT-hook 1; KLF6, Kruppel-like factor 6; AT_1_-R, Ang II receptor type 1; STAT3, signal transducers and activators of transcription-3.

To validate our cell culture data *in vivo*, we assessed the expression of AGT in K-Ras^LA2-G12D^ mice, a model of lung cancer expressing oncogenic K-Ras. K-Ras^LA2-G12D^ mice are heterozygous for the K-Ras G12D mutation, and 100% of mice develop lung adenocarcinomas as early as 3 to 4 months of age with a histopathology very similar to human disease (46). Thus, it is a well-established mouse model to study lung cancer development. We show that AGT mRNA was significantly upregulated in the lungs of K-Ras^LA2-G12D^ mice, as compared with control C57Bl6 mice (Fig. 10*A*). To test the protumorigenic properties of the AT_1_-R *in vivo*, we performed intraperitoneal (IP) injections of either vehicle DMSO or losartan in K-Ras^LA2-G12D^ and lung tumor development in these mice was quantified after 2 weeks. We find that the number of surface lung tumors was significantly reduced in the losartan-treated K-Ras^LA2-G12D^ group, as compared with mice treated with DMSO (Fig. 10*B*). Consistent with the data obtained in lung cancer cells (Fig. 9*C*), the level of phospho(Tyr705)-STAT3 in the lungs of K-Ras^LA2-G12D^ mice was downregulated by the treatment with losartan (Fig. 10*C*). Finally, we show that AGT (Fig. 10*D*) and HMGA1 (Fig. 10*E*) mRNAs were upregulated while KLF6 mRNA (Fig. 10*F*) was downregulated in human lung adenocarcinoma, as demonstrated by The Cancer Genome Atlas analysis. Thus, activation of the AT_1_-R–mediated pathway occurs in lung cancer *in vivo* and has potential clinical significance.

## Discussion

The AGT/Ang II signaling is activated by a drop of blood pressure and occurs through a multiorgan signaling that is initiated by the liver-dependent production of AGT, followed by the conversion of AGT to Ang I by the kidney-mediated release of renin and Ang I to Ang II by ACE. Our findings demonstrate for the first time that Ang II can be produced in a cell-autonomous, intrinsic and renin/ACE-independent manner after oncogene activation. We show that oncogenic K-Ras activates the AGT gene promoter and upregulates AGT protein expression in both normal and NSCLC cells but through distinct transcriptionally regulatory mechanisms (Fig. 10*G*). In normal cells (fibroblasts and NHBE cells), KLF6 controls AGT gene promoter activation while HMGA1 is a major regulator of AGT transcription in NSCLC cells. This differential activation is functionally associated with the different levels of expression of these two transcription factors in normal cells expressing K-Ras^G12V^ and NSCLC cells carrying oncogenic K-Ras. More precisely, KLF6 is upregulated by the expression of K-Ras^G12V^ in normal cells. Conversely, HMGA1 is upregulated in NSLCL cells, in which KLF6 expression is virtually lost. These data are supported by our expression analysis in humans showing upregulation of HMGA1 and downregulation of KLF6 in lung adenocarcinoma, as compared with normal lung tissue. Thus, it appears that NSCLC cells maintain AGT expression by upregulating HMGA1 expression to compensate for the loss of KLF6. These data are consistent with the loss of heterozygosity that has been described in the KLF6 gene locus (47) and with the correlation that exists between overexpression of HMGA1 and metastasis and poor survival (48) in patients with lung cancer. Moreover, because overexpression of K-Ras^G12V^ in normal cells, K-Ras^G12S^ in A549, and K-Ras^Q61H^ in H460 all upregulated AGT expression, these results suggest that the ability of oncogenic K-Ras to upregulate AGT expression does not depend on one specific oncogenic amino acid substitution at a given site but rather relies on oncogenic properties of K-Ras that are common to multiple mutant forms of K-Ras.

How is AGT converted to Ang II after oncogenic K-Ras activation? Our data show that chymase is the main enzyme mediating the K-Ras^G12V^–induced synthesis of Ang II in normal cells. In contrast, oncogenic K-Ras–expressing NSCLC cells rely on TPA and cathepsin D and they do not express chymase. Thus, similarly to the oncogenic K-Ras–mediated transcription of AGT, the K-Ras^G12V^–dependent conversion of AGT to Ang II occurs through different mechanisms in normal and NSCLC cells (Fig. 10*G*). Importantly, the ability of oncogenic K-Ras to promote synthesis of Ang II in normal and NSCLC cells occurs when cells are cultured in petri dishes and in serum-free medium, suggesting that the process occurs in a cell-autonomous and intrinsic manner. Moreover, both normal and NSCLC cells do not express either renin or ACE, indicating that oncogenic K-Ras–mediated synthesis of Ang II is renin- and ACE-independent. These findings highlight the existence of a novel signaling axis linking oncogenic K-Ras to the cell autonomous upregulation of AGT expression and synthesis of Ang II, which is independent of the extrinsic Ang II pathway that regulates blood pressure. One possible caveat to this conclusion is the fact that the ELISA kit used in our experiments to detect Ang II can theoretically recognize also Ang III and Ang IV. Thus, we cannot rule out that these other angiotensin forms can play a role in the oncogenic K-Ras–mediated signaling identified in our studies. However, because (1) we show in Figure 4*C* that inhibition of the enzymes that can convert AGT to Ang II significantly inhibited the K-Ras–induced upregulation of the angiotensin form(s) detected using this ELISA kit, (2) the major physiological role of Ang III is in the brain, and (3) Ang IV does not bind to the AT_1_-R and our data show a key role of AT_1_-R activation in K-Ras–mediated signaling, we believe that Ang II is the major form of angiotensin produced in normal and lung cancer cells after K-Ras expression.

In this study, we also investigated the functional significance of the oncogenic K-Ras–induced AGT activation and Ang II synthesis in both normal and cancer cells. We describe how this signal pathway has double-edge sword features. On the one hand, the synthesis of Ang II induced by oncogenic K-Ras induces premature senescence, an antitumorigenic mechanism, in normal cells. On the other hand, oncogenic K-Ras expression in NSCLC cells promotes proliferation and certain transformation properties through Ang II-mediated signaling. Blockade of Ang II signaling by AT_1_-R antagonists inhibits OIS in normal cells, proliferation and anchorage-independent growth of NSCLC cells, and lung tumor development in mice. These data contribute to explain the pleiotropic functions of K-Ras, which is known to induce OIS in nontransformed cells but to mediate the transformation properties of cancer cells, such as NSCLC cells, after senescence is evaded. Thus, our findings indicate the existence of an oncogene-activated local angiotensin system with either antitumorigenic or protumorigenic properties depending on the cellular context in which it is activated (Fig. 10*G*).

The concept that the lung possesses the machinery necessary for local Ang II synthesis is supported by data showing that treatment with bleomycin promotes AGT expression and Ang II synthesis in normal alveolar epithelial cells, A549 lung cancer cells, and lung myofibroblasts in cell culture studies (49, 50, 51). Similarly, activation of a local angiotensin system in the lung was described in a bleomycin-induced pulmonary fibrosis model *in vivo* (52). Interestingly, bleomycin can also induce senescence in fibroblasts and epithelial cells (53), and senescence has been causally linked to pulmonary fibrosis (54). Thus, based on our data showing that the intrinsic production of Ang II induces senescence in normal cells, it is possible that bleomycin induces senescence through Ang II–mediated signaling and that senescence induced by Ang II synthesis contributes to bleomycin-induced lung fibrosis.

How is increased Ang II synthesis linked to premature senescence after oncogenic K-Ras expression in normal cells? Activation of NOX enzymes promotes ROS generation (29) and oncogenic K-Ras promotes senescence in a ROS-dependent manner (7, 55, 56). Our data identify a novel mechanism through which oncogenic K-Ras promotes ROS-mediated senescence. We find that activation of the AT_1_-R by Ang II, upon K-Ras^G12V^–mediated upregulation of AGT, activates NOX2, which leads to cellular senescence through oxidative stress (Fig. 10*G*). We find that AT_1_-R interacts with caveolin-1 and that their interaction is not dependent on K-Ras^G12V^ expression. NOX2 is not localized in caveolar membranes under resting conditions, but expression of oncogenic K-Ras promotes the interaction of NOX2 with caveolin-1, suggesting that NOX2 moves to caveolar membranes after oncogene stimulation. Consistent with these data, AT_1_-R interacts with NOX2 only in cells expressing K-Ras^G12V^ and such interaction is lost in cells lacking caveolin-1. Together, our data show that coupling of the AT_1_-R with NOX2 is promoted by oncogenic K-Ras and occurs in caveolar membranes. Interestingly, we have previously shown that the ability of K-Ras^G12V^ to induce premature senescence is inhibited in caveolin-1 null cells (39). Based on our current findings, we speculate that the inability of the AT_1_-R to couple with NOX2 in the absence of caveolin-1 expression is one of the molecular mechanisms explaining reduced OIS in caveolin-1–lacking cells. Moreover, we have previously reported that lung tumorigenesis induced by K-Ras^G12D^ is enhanced in caveolin-1 null mice (39). Given the antitumorigenic properties of cellular senescence, we speculate that a lack of oncogenic K-Ras–initiated and caveolin-1–mediated ROS production by NOX2, after the activation of the AT_1_-R by Ang II in caveolin-1 null mice, contributes to the evasion of OIS and the enhanced lung tumorigenesis that we have observed in these mice.

Our data show that a molecular switch occurs once OIS is bypassed. Activation of the AT_1_-R by Ang II in oncogenic K-Ras–expressing NSCLC cells is converted to a protumorigenic signaling in which the AT_1_-R is coupled to STAT3 activation in a manner that is independent of NOX activation (Fig. 10*G*). In fact, overexpression of the AT_1_-R activates STAT3 while either the pharmacological blockade of the AT_1_-R or downregulation of HMGA1, the transcriptional activator of the AGT gene promoter in NSCLC cells, inhibits activation of STAT3 in NSCLC cells. STAT3 is being investigated as potential target for cancer treatment. Inhibition of STAT3 pathway activation can induce apoptosis in cancer cells (57, 58). Interestingly, we find that treatment with the AT_1_-R antagonist losartan enhanced apoptosis induced by a STAT3 pathway small-molecule inhibitor (SID 864,669) (data not shown). Because activation of the AT_1_-R by Ang II is upstream of STAT3, these data suggest that blockade of the AT_1_-R sensitizes NSCLC cells to STAT3 pathway inhibition and that a combination therapy consisting of a STAT3 pathway inhibitor and an AT_1_-R antagonist might represent a potential novel and alternative therapeutic approach for cancer treatment. In addition, since our data show that inhibition of TPA and cathepsin D, the enzymes that mediate Ang II synthesis in NSCLC cells, has antitumorigenic properties, one might envision the development of novel therapies in which inhibition of these enzymes is tested in combination with either AT_1_-R or STAT3 pathway inhibitors.

Our findings support preclinical studies that have emerged over the last few years showing how the Ang II/AT_1_-R pathway controls most hallmarks of cancer (59, 60). Moreover, our data support multiple clinical studies that have revealed beneficial effects of inhibitors of the Ang II/AT_1_-R signaling in a variety of cancers, including NSCLC (61, 62, 63). In these studies, inhibitors of the renin-angiotensin system provided better outcomes in patients with NSCLC who received platinum-based chemotherapy. Because our findings demonstrate an important protumorigenic role of local K-Ras/AGT/Ang II/AT_1_-R signaling in NSCLC cells, we speculate that the beneficial effects of AT_1_-R blockers in these studies might be the consequence, at least in part, of their ability to block local AT_1_-R signaling that is mediated by oncogenic K-Ras in transformed lung cancer cells.

Finally, because our data also show that local Ang II/AT_1_-R signaling has antitumorigenic properties in nontransformed cells, we speculate that the use of AT_1_-R inhibitors might potentially have detrimental properties in nontransformed cells in which mutation of oncogenic K-Ras has already occurred by promoting evasion from cellular senescence. One might envision a scenario in which mutant K-Ras–carrying individuals who take AT_1_-R blockers are initially more likely to develop cancerous nodules, as compared with individuals who do not take any medications targeting Ang II signaling, because of the bypassing of OIS. However, disease progression is inhibited among individuals who remain on AT_1_-R blockers vs. no medication, once nodules have developed, thanks to the ability of AT_1_-R blockers to inhibit the protumorigenic function of AT_1_-R–mediated signaling in transformed cells of cancerous nodules. Additional retrospective and future prospective studies are necessary to fully address this hypothesis.

## Experimental procedures

### Materials

Antibodies were obtained from the following sources: anti-AGT immunoglobulin G (IgG), anti-HMGA1 IgG (D6A4), anti-STAT3 IgG (4904), and anti–phospho-STAT3(Tyr705) (9131) IgG were from Cell Signaling Technology (Danvers, MA); anti-KLF6 IgG (9A2) was from Thermo Fisher Scientific (Waltham, MA); anti-HA IgG (16B12) was from BioLegend, Inc (San Diego, CA); anti-caveolin-1 IgG (pAb N-20), anti-β-actin (mAb C4), anti-p21 (F-5), and anti-cMyc (mAb 9E10; pAb A-14) were from Santa Cruz Biotechnology (Santa Cruz, CA); anti-p16 ARC (mAb EP1551Y) and anti-NOX2 (mAb EPR6991) were from Abcam (Cambridge, MA). Scrambled, human KLF6 (s3376) and human HMGA1 (s6667) siRNAs were obtained from Life Technologies (Frederick, MD). pCXB2-HA-AT1R-YFP was a gift from Yusuke Ohba (Addgene plasmid # 101659; http://n2t.net/addgene:101659; RRID:Addgene_101659) (64). The G12V substitution is one of the most common K-Ras mutations found in lung cancer. Human K-Ras^G12V^ cDNA was cloned into the lentiviral vector pLVX using standard cloning techniques. Lentivirus was generated by cotransfecting lentiviral vectors with pMD2G and pSpAX2 into 293T cells using the calcium phosphate method. Losartan, Irbesartan, DPI, VAS2870, chymostatin D, antipain, PPACK, and ruxolitinib were from Sigma-Aldrich (St Louis, MO). All other biochemical reagents were of the highest available purity and were commercially obtained.

### Cell culture

MEFs were cultured in Dulbecco’s modified Eagle's medium (DMEM), supplemented with 2-mM glutamine, 100 U/ml penicillin, 100 μg/ml streptomycin, and 10% fetal bovine serum. WI-38 human diploid fibroblasts were purchased from the American Type Culture Collection and cultured in Eagle’s Minimal Essential Medium supplemented with 2-mM glutamine, 100 U/ml penicillin, 100 μg/ml streptomycin, and 10% fetal bovine serum. NHBE cells were obtained from Lonza and cultured in SAGM BulletKit media. A549 (carrying G12S K-Ras) and H460 (carrying Q61H K-Ras) cells were cultured in Ham’s F12 medium, supplemented with 2-mM glutamine, 100 U/ml penicillin, 100 μg/ml streptomycin, and 10% fetal bovine serum.

### Primary culture of mouse embryonic fibroblasts

Pregnant mice were sacrificed, and E13.5 embryos were isolated; the head and liver were separated and discarded. The remaining embryonic tissue was minced with a razor blade and trypsinized in a 10-cm dish at 37 °C for 5 min. Next, 20 ml of DMEM supplemented with 10% fetal bovine serum and glutamine, and antibiotics (penicillin and streptomycin) were added to the dish, and the cells were incubated at 37 °C. After 24 h, the medium was replaced and cells were cultured for an additional 48 h. When confluent, MEFs were split 1:4. After 48 h, the MEFs (representing passage 1) were frozen. Experiments were performed with the use of MEFs at passage 1 or 2.

### Immunoblotting

Cells were collected in the boiling sample buffer. Cellular proteins were resolved by SDS-PAGE (12.5% acrylamide) and transferred to Amersham Protran 0.2-μm nitrocellulose blotting membrane (GE Healthcare Life Sciences, Marlborough, MA). Blots were incubated for 1 h 15 min in TBST (10-mM Tris-HCl, pH 8.0, 150-mM NaCl, 0.2% Tween 20) containing 2% powdered skim milk and 1% bovine serum albumin. After three washes with TBST, membranes were incubated overnight with the primary antibody and washed three more times with TBST. Blots were then incubated for 1 h 15 min with horseradish peroxidase–conjugated goat anti-rabbit/mouse IgG. Bound proteins were detected using an ECL detection kit (Pierce, Rockford, IL) according to the manufacturer’s protocol. Equal protein loading was assessed by Ponceau S staining. Quantification of protein expression was performed from three biological replicates. Bands were scanned, and their density was quantified for statistical analysis. The mean ± SE is shown in *italics* for each sample within the displayed representative blot. An ∗ indicates that differences are statistically significant, as calculated using Student’s *t*-test.

### Generation of human AGT promoter reporter constructs

The human AGT gene promoter used in these studies [AGT(−1000 bp)] consists of the first 1000 bp from the starting codon. It was generated by PCR with the following primers: ggCCgg**ggTACC**TCCTgggTAATTTCATgTCTg (forward) and ggCCgg*AAgCTT*AgAACAACggCAgCTTCTTCC (reverse). AGT promoter deletion mutants were generated by PCR using the following reverse primers: AGT (−1000/−250 bp), ggCCgg*AAgCTT*CCgAAgTTTgCAggAgTCggg; AGT (−1000/−500 bp), ggCCgg*AAgCTT*AgAgCCTACCCTgTgCAAAgg; AGT (−1000/−750 bp), ggCCgg*AAgCTT*TTTTCCTATAggTAAgACTTg. Kpn I binding site is shown in bold; Hind III sites are in italic. AGT promoter mutants Δ1, Δ2, and Δ3, which lack KLF6 binding sites at positions −445 bp, −354 bp and −15 bp, respectively, were generated by PCR. All PCR products were cloned with KpnI/Hind III into the luciferase-based vector pTA-luc in which the TATA box was removed (Clontech, Mountain View, CA).

### Luciferase reporter assay

Cells were seeded in 60-mm dishes at 270,000 per dish. The following day, cells were transiently transfected, using a modified calcium-phosphate precipitation method, with 2 μg of the human AGT promoter luciferase reporter constructs and 1 μg of a β-galactosidase–expressing construct. Twenty-four hours after transfection, cells were rinsed twice with PBS and incubated in the complete medium for an additional 48 h. Cells were then lysed in 400 μl of the extraction buffer; 200 μl was used to measure luciferase activity, and 100 μl was used to measure β-galactosidase activity as previously described (65). Three independent experiments were performed in triplicate for each condition.

### ChIP assays

MEFs were infected with either the empty vector pLVX or pLVX-K-Ras^G12V^. After 7 days, ChIP was performed with the ChIP assay kit from Millipore (Burlington, MA). Briefly, cells were cross-linked with 1% formaldehyde for 10 min at 37^o^C. Cells were washed with cold PBS, harvested, and the DNA-fragmented into ∼250- to 1000-bp fragments by sonication using the Cole Parmer Ultrasonic Processor sonicator (model CPX 750). ChIP lysates were diluted and precleared with salmon sperm DNA–protein A-agarose beads for 30 min at 4^o^ C. One-tenth of the supernatant was taken as input DNA for PCR to show equal chromatin content before immunoprecipitation. The rest of the supernatant was then incubated overnight at 4^o^ C with anti-KLF6 IgGs and salmon sperm DNA protein A-agarose beads. After washes, PCR was performed in the exponential linear zone of amplification with the following primers, which flank KLF6 sites in the AGT gene promoter: gcttgtgtgttttccccagtg (forward) and aagaggccacagggacatgca (reverse). A representative gel is shown.

### Quantification of Ang II expression

Ang II levels in the conditioned medium and in cell lysates were measured using the Angiotensin II EIA kit (RAB0010) from Sigma-Aldrich (St Louis, MO), according to the manufacturer’s reccomendations.

### SA-β-gal activity assay

SA-β-gal activity was measured using the senescence β-galactosidase staining kit according to the manufacturer’s protocol (Cell Signaling Technology, Danvers, MA). The average percent senescence was calculated from quantification of total cells and senescent cells in 10 fields of view per condition, using an upright epifluorescent Leica microscope. Representative images of microscope fields are shown.

### Transfections of siRNA

The siRNAs used in our studies are *Silencer Select siRNAs* from Thermo Fisher Scientific, which include locked nucleic acid chemical modifications that reduce off-target effects by up to 90%. For each experiment, we have used, as negative control, a *Silencer Select negative control*, a nontargeting control with no significant similarity to mouse or human gene sequences, which are also chemically modified for reduced off-target effects. siRNA was introduced into cells using Lipofectamine RNAiMax Reagent from Life Technologies (Frederick, MD) according to the manufacturer’s protocol, using 45 pmol per well of 6-well culture plates.

### Quantification of hydrogen peroxide

Hydrogen peroxide was quantified using the Amplex Red Hydrogen Peroxide Assay kit (A22188) from Thermo Fisher Scientific (Waltham, MA), according to the manufacturer’s reccomendations.

### RNA isolation and RT-PCR

Cells were collected and total RNA was isolated using the RNeasy Mini kit from Qiagen (Valencia, CA). Equal amounts of RNA were treated with RNase-free DNase and subjected to reverse transcription using the Advantage RT-for-PCR kit from Clontech (Mountain View, CA), according to the manufacturer’s protocol. PCR was then performed for each gene studied in its linear zone of amplification.

### Immunoprecipitation

Cells were washed twice with PBS and lysed for 30 min at 4 °C in a buffer containing 10-mM Tris, pH 8.0, 0.15 M NaCl, 5-mM EDTA, 1% Triton X-100, and 60-mM octyl glucoside. Samples were precleared for 1 h at 4 °C using protein A-Sepharose (10 μl; slurry, 1:1) and subjected to overnight immunoprecipitation at 4 °C using the intended antibody and protein A-Sepharose (30 μl; slurry, 1:1). After three washes with the immunoprecipitation buffer, samples were separated by SDS-PAGE (12.5% acrylamide) and transferred to nitrocellulose. Then, blots were probed with the intended antibody. Experiments were performed three independent times, and representative images are shown.

### BrdU incorporation assay

Cell proliferation was measured and quantified using the Cell Proliferation ELISA, BrdU (colorimetric) kit (Roche, Indianapolis, IN). The kit was used according to the manufacturer’s protocol. Cells were incubated with BrdU labeling solution overnight at 37 °C. The absorbances were measured at 370 nm after substrate incubation using an ELISA plate reader.

### Growth in soft agar

Cells (5 x 10^4^) were suspended in 3 ml of complete medium and 0.33% SeaPlaque low-melting temperature agarose. These cells were plated over a 2-ml layer of solidified complete medium and 0.5% agarose and allowed to settle to the interface between these layers at 37^o^C. After 30 min, the plates were allowed to harden at room temperature for 30 min before returning to 37^o^ C. After 10 days, colonies were photographed under low magnification (5x). The colonies in 60 randomly chosen fields from three independent plates were counted.

### K-Ras^LA2-G12D^ mouse model of lung cancer

K-Ras^LA2-G12D^ mice are heterozygous for the K-Ras G12D mutation and 100% of mice develop lung adenocarcinomas as early as 3 to 4 months of age with a histopathology very similar to human disease (46). Thus, it is a well-established mouse model to study lung cancer development. Animal work was approved under the University of Pittsburgh IACUC protocol number 19044828.

### Losartan injections in mice and lung tumor counts

K-Ras^LA2-G12D^ mice (2-week-old) were subjected to daily IP injections of losartan (50 mg/kg; 100 μl in DMSO). DMSO was injected as control. IP injections of losartan (50 mg/kg) have been shown to inhibit Ang II–dependent and AT_1_-R–mediated physiological activities in mouse studies (66, 67). After 14 days, mice were euthanized and lungs were perfused with PBS and inflated with optimal cutting temperature compound. After 5 min, lungs were extracted and surface lung tumors were counted.

### Statistical analysis

Studies were performed in triplicates using three biological replicates to achieve statistically significant differences. The average ± SEM is shown. Significance was calculated using Student’s *t*-test.

## Data availability

All of the data are contained within the manuscript.

## Conflict of interest

The authors declare that they have no conflicts of interest with the contents of this article.
