# Supplementary material for: Cell autonomous angiotensin II signaling controls the pleiotropic functions of oncogenic K-Ras
Source: J Biol Chem. 2021 Jan 8;296:100242. doi: 10.1074/jbc.RA120.015188 (PMC7948762; doi:10.1074/jbc.RA120.015188)

Figure S1. Oncogenic K-Ras transcriptionally activates the angiotensinogen gene promoter in NHBE cells. (*A*) Schematic representation of the angiotensinogen gene promoter deletion mutants used in our studies. (*B*) NHBE cells were infected with either vector alone (pLVX) or pLVX-K-Ras^{G12V}. After 3 days, cells were transfected with a luciferase-carrying vector (pTA-LUC) under the control of the first 1,000bp of the angiotensinogen gene promoter (-1,000/-1) and the following promoter deletion mutants: (-1,000/-250), (-1,000/-500) and (-1,000/-750). After 48 hours, cells were collected and luciferase activity was measured. (*C*) Schematic diagram showing point mutations created to individually eliminate each of the three KLF6 binding sites found on the AGT gene promoter (MUT 1, MUT 2 and MUT 3). (*D*) NHBE cells were infected with either pLVX or K-Ras^{G12V}. After 3 days, cells were transfected with either control siRNA (Ctl siRNA) or siRNA against KLF6 (KLF6 siRNA). After 4 days, cells were collected and RT-PCR analysis was performed using primers specific for AGT. RT-PCR for GAPDH was performed as internal control. Values in *B* represent means ± SEM. Statistical comparisons were made using the student's t-test. $^*P<0.005$.

Figure S2. Oncogenic K-Ras promotes Ang II synthesis in a cathepsin D- chymase- and TPA-dependent manner in NHBE cells. *(A)* NHBE cells were infected with a lentiviral vector expressing K-Ras^{G12V}. Infection with pLVX alone was used as control. After 7 days, angiotensin II (Ang II) was quantified in the conditioned medium derived from pLVX- and pLVX-K-Ras^{G12V}-expressing NHBE cells. *(B)* NHBE cells were infected with either pLVX or K-Ras^{G12V}. After 7 days, expression of cathepsin D, chymase, tissue plasminogen activator (TPA), renin, and angiotensin-converting-enzyme (ACE) mRNAs was quantified by RT-PCR analysis. Quantification of GAPDH was done as internal control. (*C*) Renin mRNA expression in the kidney and ACE mRNA expression in the lung was determined as positive control. *(D)* K-Ras^{G12V} was over-expressed in NHBE cells for 7 days in the presence of antipain (5μM), chymostatin (10μM) or PPACK (10μM). Treatment with DMSO was performed as control. Angiotensin II (Ang II) was quantified in the conditioned medium of these cells. Values in *A* and *D* represent means ± SEM. Statistical comparisons were made using the student's t-test. $^*P<0.005$.

Figure S3. Inhibition of either AT_1-R activity or Ang II synthesis impair oncogene-induced senescence. (*A-D*) NHBE cells were infected with a lentiviral vector (pLVX) expressing K-Ras^{G12V}. Infection of NHBE cells with pLVX was performed as control. Cells were cultured for 10 days in the presence of either losartan (5μM) or chymostatin (10μM). Treatment with DMSO served as control. In *A* and *B*, cells were subjected to senescence-associated β-galactosidase (SA-β-gal) staining. Quantification is shown in *A*, representative images are shown in *B*. Scale bar = 100 μm. In *C*, cell lysates were subjected to immunoblotting analysis using antibody probes specific for the senescence markers p16 and γ-H2A.X. In *D*, SA cell morphology was quantified from a total of at least 300 cells from 10 randomly chosen fields. Values in *A* and *D* represent means ± SEM. Equal protein loading was assessed by Ponceau S staining in *C*. Blots are representative of three independent experiments. Mean ± SE is shown for each sample. Statistical comparisons were made using the student's t-test. $^*P<0.005$.

Figure S4. Downregulation of the AT_1-R inhibits K-Ras^{G12V}-induced ROS generation. K-Ras^{G12V} was expressed in WT mouse embryonic fibroblasts. Infection of MEFs with the empty pLVX vector served as control. After 3 days, cells were transfected with either control siRNA (Ctl siRNA) or AT_1-R siRNA. Cells were cultured for 4 additional days. In *A*, downregulation of AT_1-R was quantified by immunoblotting analysis with anti-AT_1-R IgGs. In *B*, hydrogen peroxide levels were measured by Amplex Red assay. Values in *B* represent means ± SEM. Equal protein loading was assessed by Ponceau S staining in *A*. Blots are representative of three independent experiments. Mean ± SE is shown for each sample. Statistical comparisons were made using the student's t-test. $^*P<0.005$.

Figure S5. Downregulation of AGT expression by siRNA in H460 NSCLC cells. Antipain and chemostatin inhibit cathepsin D and TPA activity, respectively. (*A*) Schematic diagram showing two putative HMGA1 binding sites on the AGT promoter. (*B*) H460 cells were transfected with either control siRNA (Ctl siRNA) or angiotensinogen (AGT) siRNA in the presence or absence of an expression vector carrying the AGT cDNA (+AGT). After 72 hours, AGT protein expression was quantified by immunoblotting analysis using an antibody probe specific for AGT. (*C,D*) H460 cells were treated with either antipain (5μM) or PPACK (10μM) for 72 hours. Treatment with H_2O was performed as control. Cathepsin D activity was measured in antipain-treated H460 cells (C) while TPA activity was measured in PPACK-treated cells (D). (*E*) H460 cells were transfected with either control siRNA (Ctl siRNA) or HMGA1 siRNA in the presence or absence of an expression vector carrying the HMGA1 cDNA (+HMGA1). After 72 hours, cells were collected and HMGA1 protein expression was quantified by immunoblotting analysis using anti-HMGA1 IgGs. Values in *C* and *D* represent means ± SEM. Equal protein loading was assessed by Ponceau S staining in *B* and *E*. Blots are representative of three independent experiments. Mean ± SE is shown for each sample. Statistical comparisons were made using the student's t-test. *$P<0.005$.

Volonte et al.,
Figure S1

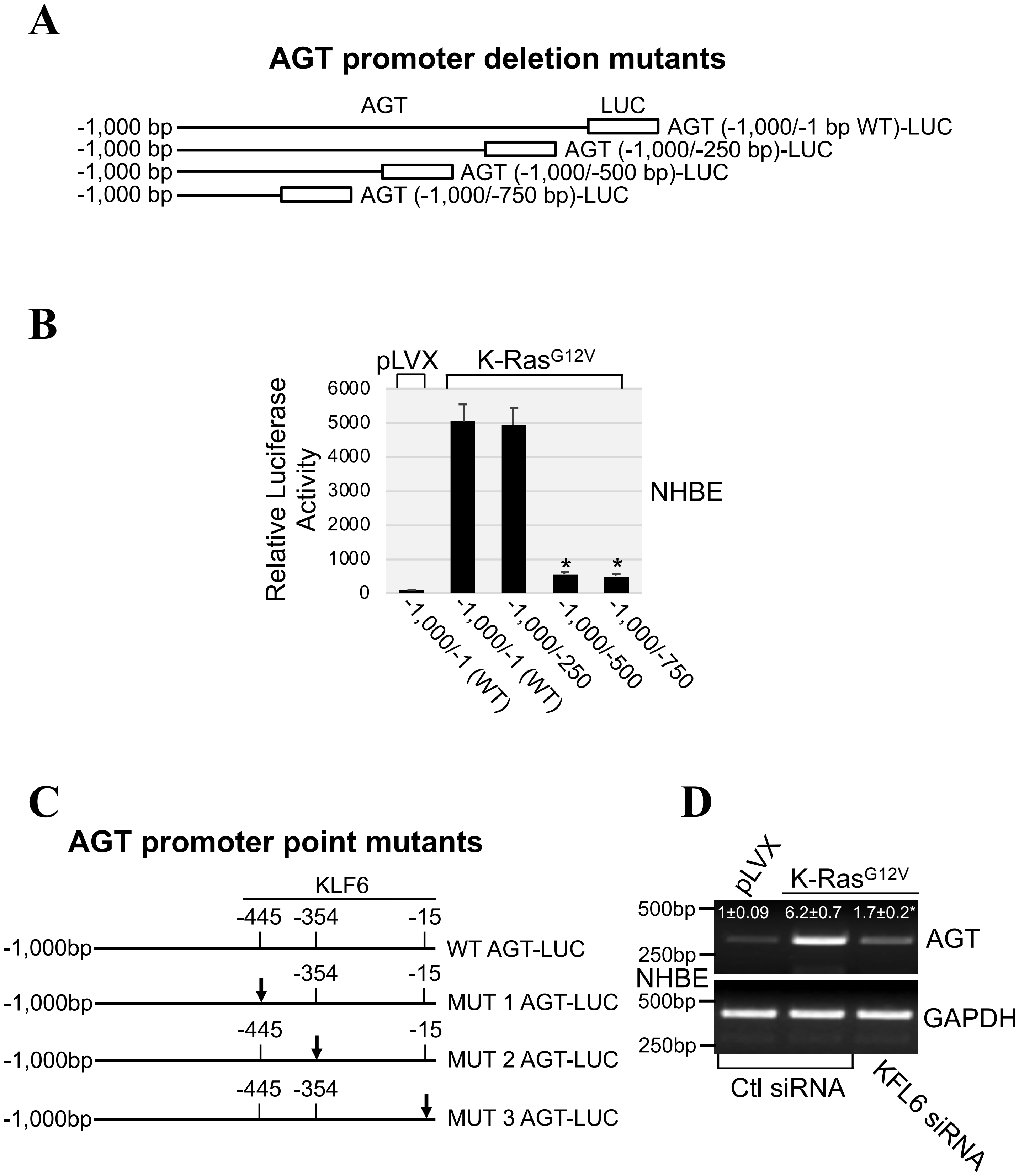

Volonte et al.,
Figure S2

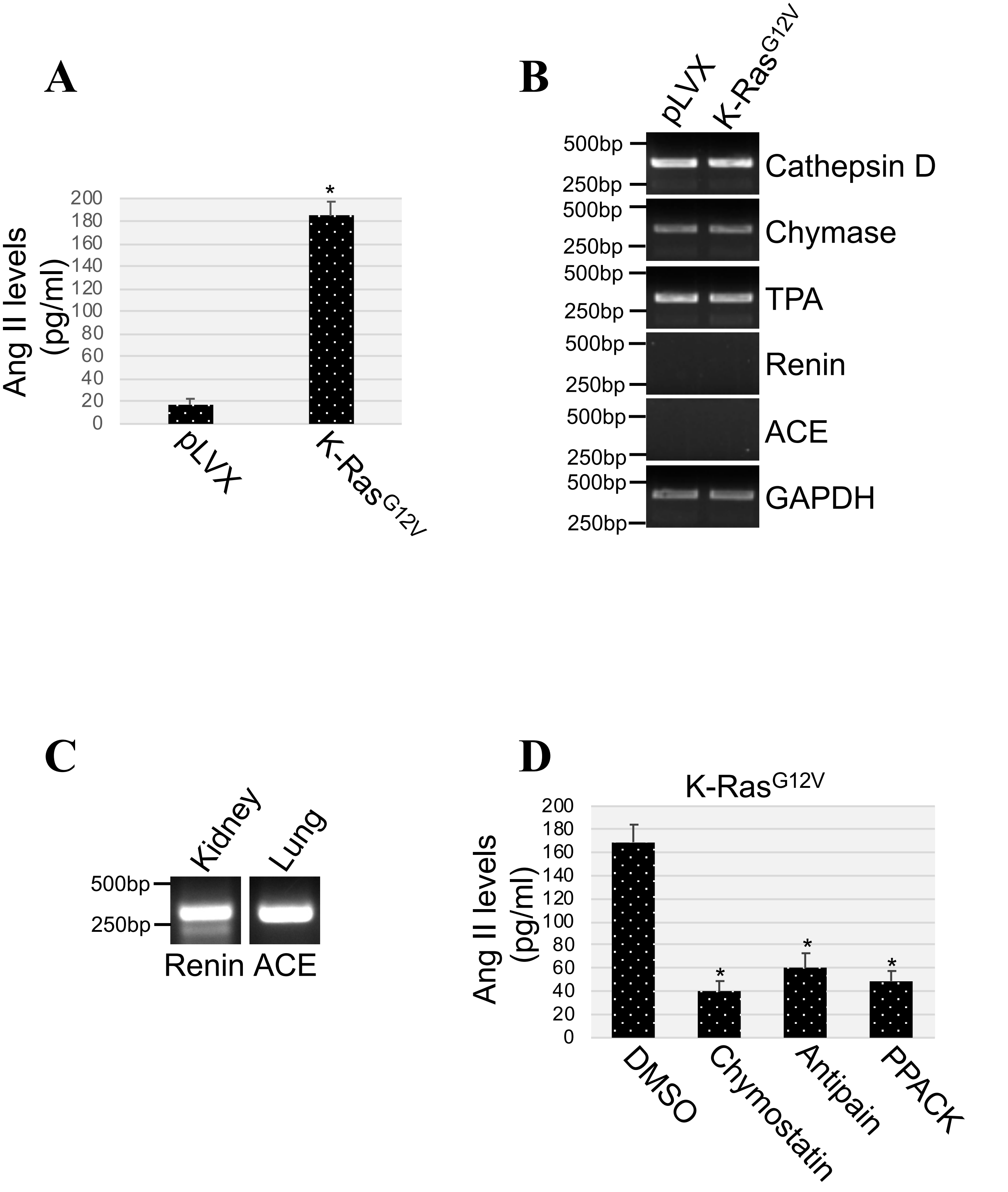

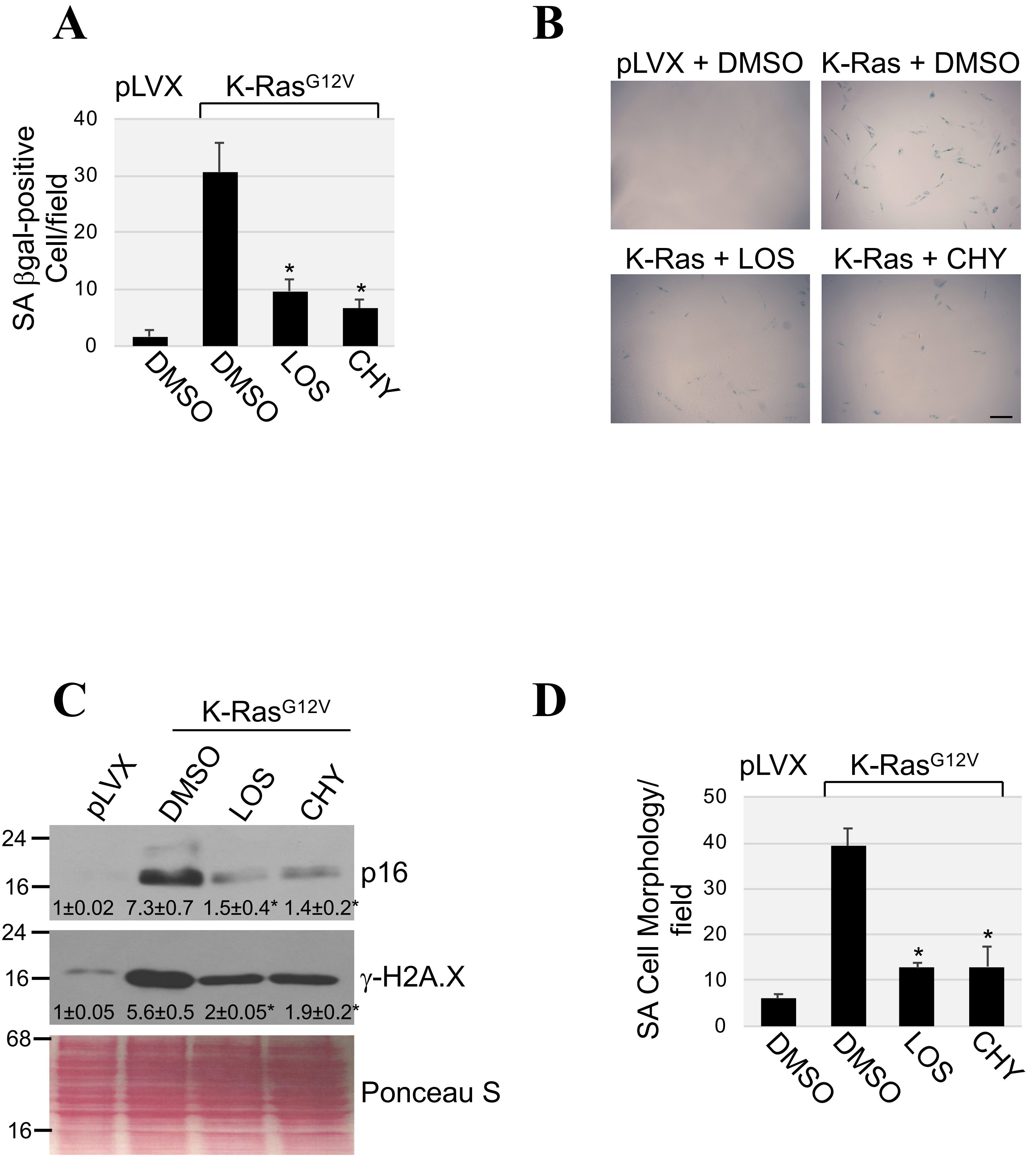
A
pLVX
K-RasG12V
SA βgal-positive Cell/field
40
30
20
10
0
*
*
DMSO
DMSO
LOS
CHY
B
pLVX + DMSO
K-Ras + DMSO
K-Ras + LOS
K-Ras + CHY
C
K-RasG12V
pLVX
DMSO
LOS
CHY
24
16
p16
1±0.02
7.3±0.7
1.5±0.4*
1.4±0.2*
24
16
γ-H2A.X
1±0.05
5.6±0.5
2±0.05*
1.9±0.2*
68
16
Ponceau S
D
pLVX
K-RasG12V
SA Cell Morphology/ field
50
40
30
20
10
0
*
*
DMSO
DMSO
LOS
CHY

Volonte et al.,
Figure S4

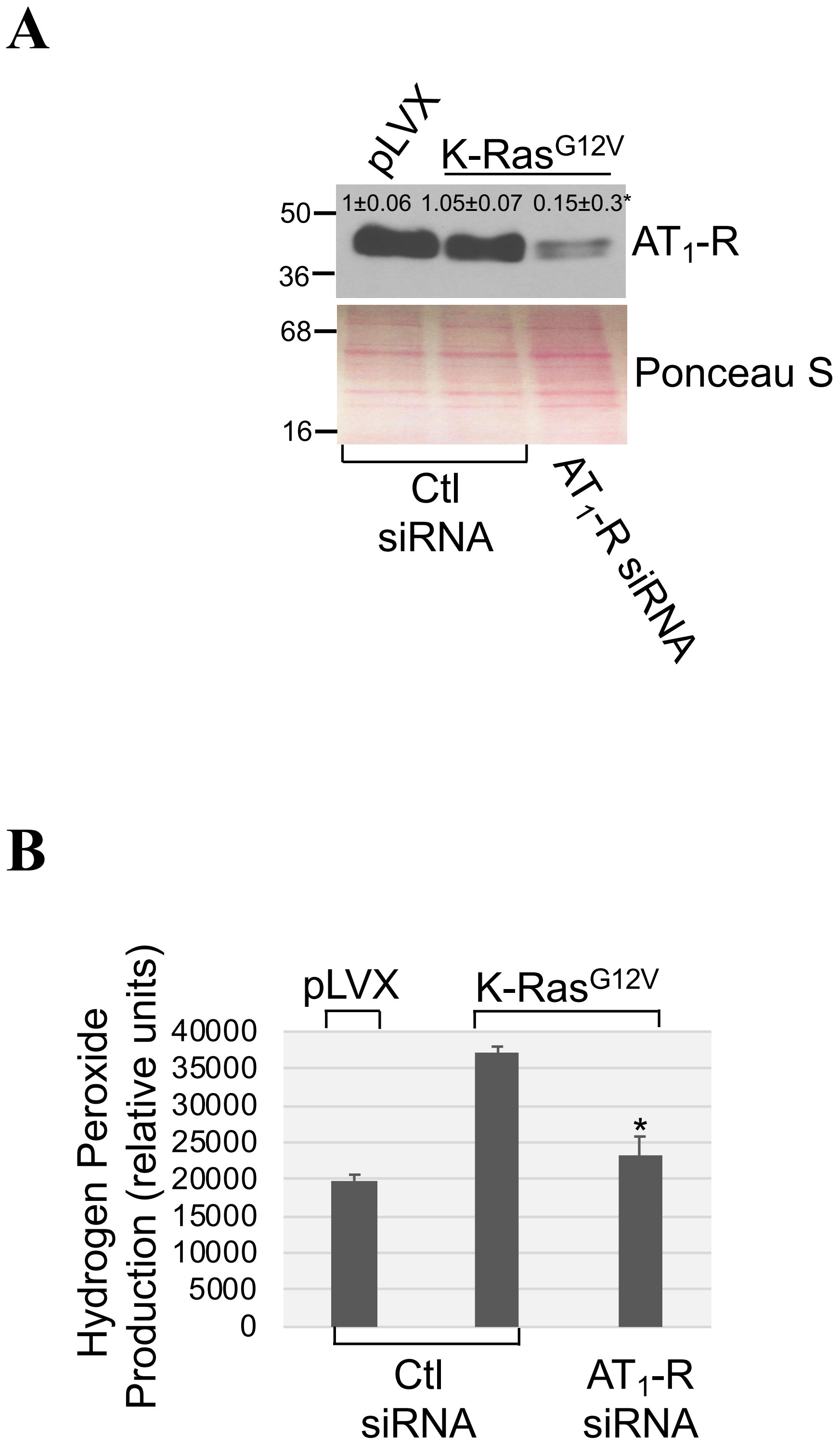

Volonte et al.,
Figure S5

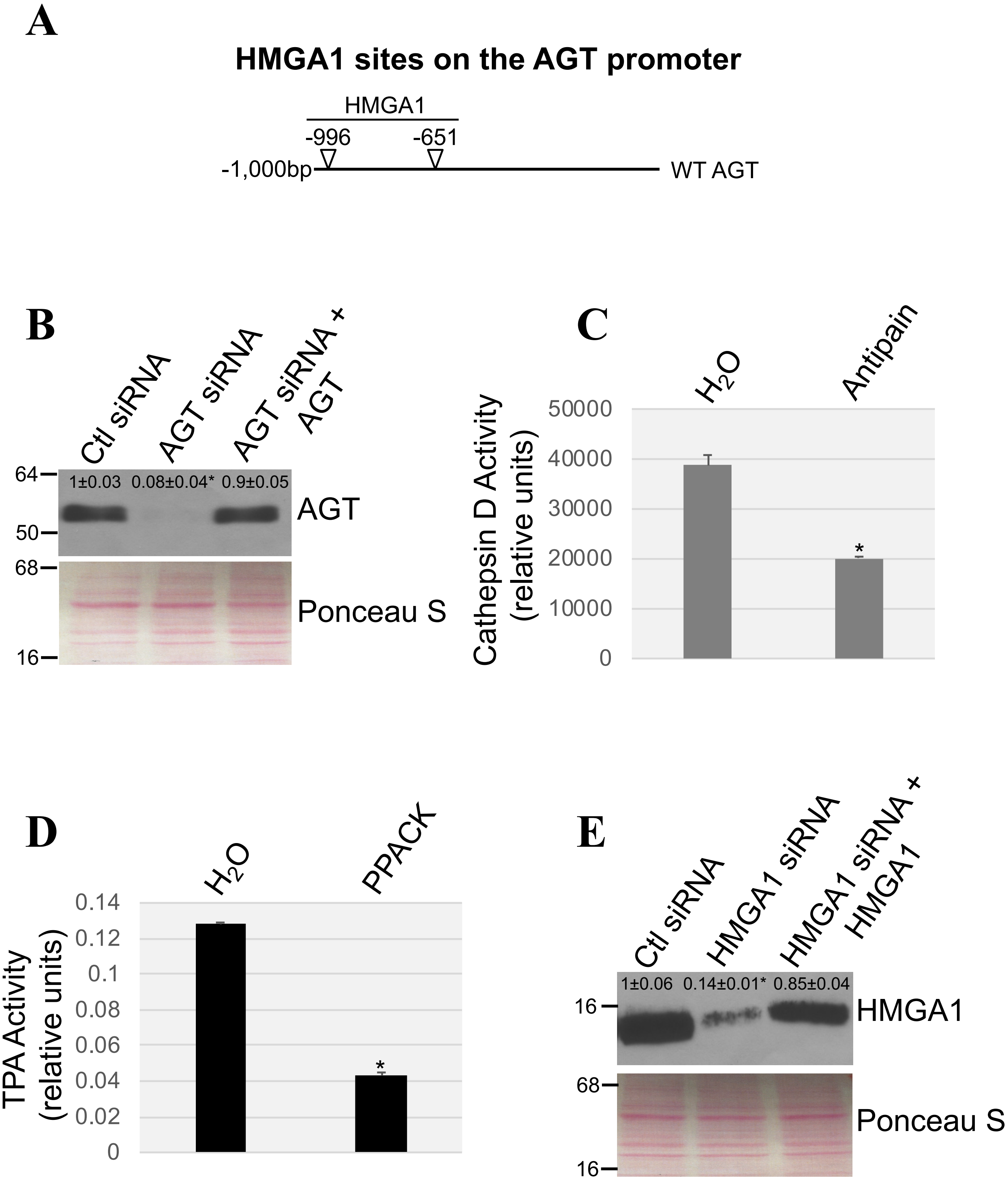

Supplement: Figures S1–S5 [file mmc1.pdf]
